# Gene expression profiles during subclinical *Mycobacterium avium* subspecies *paratuberculosis* infection in sheep can predict disease outcome

**DOI:** 10.1038/s41598-019-44670-w

**Published:** 2019-06-03

**Authors:** Auriol C. Purdie, Karren M. Plain, Douglas J. Begg, Kumudika de Silva, Richard J. Whittington

**Affiliations:** Farm Animal Health, Sydney School of Veterinary Science, Faculty of Science, University of Sydney, Sydney, Australia

**Keywords:** Transcriptomics, Infection

## Abstract

Paratuberculosis in ruminants is caused by infection with *Mycobacterium avium* subspecies *paratuberculosis* (MAP) however exposure does not predetermine progression to clinical disease. The pathogenesis incorporates a subclinical phase during which MAP is capable of evading host immune responses through adaptation of host cellular immune mechanisms. Presented are results of transcriptomic analysis of Merino sheep experimentally exposed to MAP and repeatedly sampled over the subclinical phase, identifying genes consistently changed over time in comparison to unexposed controls and associated with different disease outcomes. MAP exposed sheep were classified as diseased 45% (n = 9) or resilient 55% (n = 11). Significant gene expression changes were identified in the white blood cells of paucibacillary (n = 116), multibacillary (n = 98) and resilient cohorts (n = 53) compared to controls. Members of several gene families were differentially regulated, including S100 calcium binding, lysozyme function, MHC class I and class II, T cell receptor and transcription factors. The microarray findings were validated by qPCR. These differentially regulated genes are presented as putative biomarkers of MAP exposure, or of the specified disease or resilience outcomes. Further, *in silico* functional analysis of genes suggests that experimental MAP exposure in Merino sheep results in adaptations to cellular growth, proliferation and lipid metabolism.

## Introduction

Paratuberculosis (Johne’s disease) is a chronic, progressive granulomatous enteritis of ruminants caused by *Mycobacterium avium* subspecies *paratuberculosis* (MAP). The disease is responsible for significant global economic losses^1–6^ and there are public health concerns, with associations identified between MAP and Crohn’s disease in humans^7–10^.

Transmission of MAP occurs primarily through ingestion via the faecal-oral route. Following consumption, the mycobacteria gain entry into the intestinal tract and the underlying lymphatic system via microfold (M) cells which overlie Peyer’s patches in the ileum^11–13^. This results in the activation of the immune process when the MAP are scavenged by macrophages and either survive within these cells or their antigens are presented to T lymphocytes. Understanding the early pathogenesis of paratuberculosis will contribute to determining the immune response that is required for protection.

Ovine paratuberculosis typically manifests as weight loss and is eventually fatal; however presentation of clinical signs may be delayed resulting in a sometimes-lengthy subclinical phase^11,14,15^, during which time a subset of the MAP infected sheep act as an ongoing source of dissemination through intermittent shedding of viable MAP in faecal matter^11,16,17^. The persistence of the causative agent in the environment represents a considerable challenge for the management of paratuberculosis on-farm and accurate early identification of livestock that are both subclinically infected and infectious is not currently reliable. In addition, experimental infection trials have identified that exposure to MAP does not predetermine progression to clinical disease^18,19^ and this adds a further level of complexity for management, since identification of animals resilient to MAP infection though desirable is not currently possible.

Classification of paratuberculosis is historically assigned on the basis of post-necropsy tissue culture and histology findings^11,20^. The immune profiles of the distinct disease forms of paucibacillary (tuberculoid) and multibacillary (lepromatous) paratuberculosis^11,20^ are well characterised: sheep with the paucibacilliary form are likely to have a T helper (Th)1 cell-mediated immune response, with large numbers of CD4^+^ and γδ T lymphocytes dominating lesions at the site of infection yet lesions present with few mycobacteria^11,21^. Sheep with multibacillary paratuberculosis typically present with lesions dominated by macrophages containing large numbers of mycobacteria usually coupled with a strong Th2 humoral antibody response and declining cell-mediated immunity^22–24^. These and other findings led to a hypothesis associating differential disease progression to Th1 or Th2 dominance, although this has recently been challenged in ovine paratuberculosis^25^. Any discrepancy may be due to a lack of understanding of the dynamics and complexity of host responses during the early stages of subclinical infection (discussed and reviewed by Koets *et al*.^26^). Substantial research has been conducted on experimental infection models of ovine, caprine and bovine paratuberculosis seeking to explore variations in the early subclinical immune responses associated with disease progression^27–32^ and the potential to predict or diagnose eventual disease through use of longitudinal sampling^33^. There is increasing evidence of early, predictive immune regulation occurring in MAP-exposed animals; research within this laboratory has shown changes to MAP-specific interferon gamma (IFNγ)^34^, interleukin 10 (IL-10)^34^ and proliferative responses of peripheral blood monocytic cells (PBMC)^18^ in sheep within weeks of experimental MAP exposure, leading to a potential for accurate prediction of disease outcome from responses measured during the early subclinical phase^19,35^.

The sometimes lengthy nature of subclinical ovine paratuberculosis and transitions of peripheral immune responses incorporating the T helper cell dynamics, suggest that infection with MAP might have a significant effect on host immune cell gene expression profiles. Differential gene expression has been reported in MAP infected cattle^36^ and sheep^37^ utilising methodologies that target defined pro-inflammatory or immune function-related genes. However, there is a paucity of research describing transcriptomic-based gene expression changes in MAP exposed sheep during the subclinical phase of infection. There are several excellent reports of transcriptomic analysis of clinically diseased sheep; in 2010 Smeed *et al*. reported findings of a transcriptomic study of terminal ileum sections obtained from eighteen mixed-breed and mixed-age sheep categorised by histopathology^38^. A ruminant immuno-inflammatory gene universal array comprising of 596 genes was utilised and significant differential expression of 36 genes was observed suggesting that paucibacillary and multibacillary disease in sheep have distinct molecular profiles. Several of the authors of the 2010 study have recently published the findings of a new study^39^ in which they utilise Illumina TruSeq technology and a bovine PCR array to analyse the transcriptome of the illeocaecal lymph node tissue harvested from clinically diseased mature sheep (2–4.5 years old) and found no fundamental difference in the gene expression patterns differentiating multibacillary or paucibacillary disease, with no shift from Th1 to Th2-like patterns in the mature sheep. These studies have provided invaluable insight into the effect of paucibacillary and multibacillary disease on the expression of immune response genes and provide insights into associated pathways in clinical ovine paratuberculosis.

Identifying gene-gene interactions is essential to understand disease susceptibility and to detect genetic architectures underlying complex diseases. In the case of paratuberculosis, analysis of consistent immunopathogenomic changes in the subclinical stage may identify molecular determinants responsible for MAP exposed sheep succumbing to differential disease trajectories in comparison to those that appear to recover from infection. The key to achieving meaningful findings from disease pathogenesis-associated transcriptomic data is accurate classification of disease outcome and this is particularly important in the analysis of the subclinical immunopathogenesis of paratuberculosis because classification of outcome must be assigned to animal groupings retrospectively^40^. Thus, the objective of this study was to utilise transcriptomic methodologies to evaluate temporally consistent gene expression changes relevant to the immunopathogenesis of subclinical paratuberculosis, with reference to the final disease outcomes in sheep, with rigorous case definitions used to enable comparisons with other studies.

## Methods

All animal procedures were approved by the Animal Ethics Committee, University of Sydney (Ethics approval N00/8-2007/3/4649) and all experiments were performed in accordance with relevant guidelines and regulations.

### Ovine Johne’s disease model

A controlled experimental infection of ovine JD was established utilising a previously validated sheep infection model^18^ and is described in detail in Begg *et al*.^41^. Briefly, 30 Merino wether lambs were sourced from a property where parental flocks were free from MAP-infection (based on OJD Market Assurance Program^42^ criteria) and absence of MAP infection was confirmed through whole-flock faecal tests^43,44^ and serum antibody ELISA (IDEXX). The animals were moved to a quarantine farm at the University of Sydney, Camden, NSW (Australia) and maintained under conventional Australian sheep farming conditions by grazing on open pasture, with un-inoculated control sheep kept in separate paddocks to the MAP-inoculated sheep. Lambs between 2 to 4 months of age were drafted into groups using systematic random sampling. The 10 lambs were grouped as un-inoculated controls. The remaining 20 lambs were challenged with three oral doses of MAP inoculum administered over a period of a month with a low passage laboratory seed stock culture of MAP sheep (S) strain Telford 9.2^45,46^. The cumulative dosage of the challenge was 3.18 × 10^9^ viable MAP.

### Ante-mortem sampling and examinations

Blood, serum and faecal samples were collected from all animals at regular intervals (4–16 weeks) post MAP exposure as described by Begg *et al*.^41,47^. Blood samples collected at 2, 10, 18, 32 and 56 weeks post exposure were processed and used for transcriptomic studies; total RNA was isolated from the white blood cells and assessed using individual Affymetrix genechips (as described below). Faecal samples were stored at −80 °C until required. Serum from the blood samples was stored at −20 °C until required. Blood samples were immediately processed to prepare white blood cells (WBC) for RNA isolation and stored at −80  °C until required.

All animals were monitored by visual inspection at least three times weekly and from 32 weeks post inoculation, all animals were weighed on a monthly to weekly basis to identify individuals with clinical disease. Regular health checks ensured that the sheep remained free of other diseases.

### Necropsy and post mortem sampling

Upon evidence of clinical disease (loss of ≥10% of body weight), or at the termination of the trial at 137 weeks (2 years and 7 months), animals were sacrificed and underwent necropsy^18^. The first clinical case was identified at 57 weeks post-exposure; all timepoints used for transcriptomic analyses were prior to the culling of any animals due to clinical disease. Gross pathological changes were assessed at necropsy and a range of samples collected for disease evaluation by histopathological examination, faecal and tissue culture and presence of MAP DNA in faeces. Results related to case definition are reported in detail in Begg *et al*.^41^. In cases where MAP inoculated sheep presented evidence of weight loss, an age-matched un-inoculated control animal was also sacrificed. All remaining animals were culled at the termination of the trial, 137 weeks post inoculation.

Euthanasia of animals and collection of tissue samples were as described by Begg *et al*.^18^. Briefly, upon necropsy faecal, blood and 12 gut tissue samples including the terminal ileum, anterior jejunum, associated lymph nodes and liver sections were harvested for faecal and tissue culture^18,48^, high throughput direct PCR assay (HTJ)^43,49^ and histopathology analysis to verify clinical disease and accurately classify disease outcome^11^. Samples were stored under appropriate conditions for future analysis.

### Case definition

Each animal was categorised using case definitions for paratuberculosis^40^. Briefly, an animal was classified as clinically diseased if there was loss of ≥10% body weight over one month^18^, MAP was cultured from tissues post necropsy and there were histopathological lesions consistent with paratuberculosis. An animal was classified as infected with MAP based on a microbiological assessment, defined by a positive culture result for MAP in the gut or associated lymphoid tissues of the animal; based on this definition 9 of the 20 MAP exposed animals were defined as infected. Further to this, the pathology of paratuberculosis (paucibacillary, multibacillary) was determined by histopathological examination for acid fast bacilli (AFB) and evidence of MAP disease-specific lesions^11^. Resilient sheep were defined as animals known to have received an infectious dose of MAP in which the infection did not establish, did not progress or remained in a dormant state, and when examined at necropsy, the infection could not be detected using standard tests such as culture of tissues or histopathology. The resilient sheep displayed evidence of exposure through early faecal shedding and/or IFNγ and IL-10 immune responses^40^ and were described as resistant by de Silva *et al*.^35^ and as survivors by Begg *et al*.^41^.

### Preparation of white blood cells (WBC)

WBC were isolated from peripheral blood samples. Briefly, 9 ml blood was collected from each sheep by jugular venipuncture into EDTA coated blood vacuette tubes. Buffy coats were isolated by centrifugation at 1455 × *g* for 20 min at 22° C and residual red blood cells were lysed using ammonium chloride (0.83% NH_4_Cl, 0.1% KHCO_3_, 0.01 M EDTA pH 7.5) and the WBC pelleted by centrifugation at 233 × g for 10 min at room temperature.

### Preparation of RNA for arrays

Total RNA was isolated from WBC samples using the RNAspin Mini RNA isolation kit (Illustra, GE Healthcare). Quantity and integrity of the isolated RNA were verified by spectrophotometry (Nanodrop) and Agilent 2001 Bioanalyser analysis (acceptable RIN number 6.0-10). The RNA samples were stored at −80 °C until required for processing.

### Transcriptomic processing to Affymetrix® GeneChip® for gene expression profiling

Transcriptomic analysis of individual sheep WBC samples were carried out at 2, 10, 18, 32 and 56 weeks post MAP exposure. Samples of all 20 of the MAP inoculated and 5 of the 10 control sheep were selected for analysis at each time point: each animal contributed 5 samples and in total 125 samples were processed using the Bovine Affymetrix® GeneChip® 3′IVT array^50^. Transcriptomic processing on array chips was carried out at the Ramaciotti Centre for Genomics (UNSW Sydney, Australia). At the time the RNA samples were hybridised to the array GeneChips®, the Bovine array was determined to contain the most comprehensive coverage. The trial ran for 2.5 years post inoculation to allow for disease progression and in the intervening time Affymetrix marketed an Ovine GeneChip® Gene 1.0 ST Expression Array. Financial constrains did not allow for reanalysis of samples by this newer array, however validation of the array findings included extensive quantitative (q)PCR analysis of samples derived from all 30 animals in the trial across all five time points. The Bovine Genome Array consists of 23,000 gene transcripts and includes approximately 19,000 UniGene clusters. Briefly, 1 µg of total RNA from each sample was reverse transcribed using a 3′ IVT Express protocol as recommended by Affymetrix and hybridised to a GeneChip.

#### Microarray data analysis

Affymetrix genechip operating software (GCOS) derived raw expression values were obtained as.CEL files and transferred to Partek Genomic Suite 7 software (Partek Inc.) for analysis. Briefly, the raw data were normalised and corrected for non-specific binding following which array data quality was confirmed by Principle Component Analysis (PCA). ANOVA was applied to compare gene changes that were consistent across all timepoints (2, 10, 18, 32 and 56 weeks post MAP exposure) in comparison to the control animals. Gene annotation was then performed. This process is described in detail below.

Specifically, all samples demonstrated characteristics of high quality cRNA (3′/5′ ratio of probe sets for the integral housekeeping genes of 1.5). The raw data were normalised using the RMA (Robust Multichip Averaging) algorithm^51^. This method retains probe-level information and applies probe-specific background correction to compensate for non-specific binding found in the chips using perfect-match (PM) probe distribution. In addition, RMA normalisation applied PM distributions across all the chips and a robust probe-set summary of the log 2 normalised probe-level data by median polishing. Array data quality was confirmed by Principle Component Analysis (PCA), which examined correlation between the data derived from the different arrays and identified potential outliers. ANOVA was then used to determine those probe sets significantly different between the defined paratuberculosis outcome variables (paucibacillary, multibacillary and resilient) compared to control sheep, consistently across all timepoints, to the exclusion of the other variables. This was followed by a Benjamini and Hochberg Multiple testing correction to reduce the false positive rate^52^. To ensure the accuracy of the results, only differentially expressed genes with a false-discovery rate (FDR) less than or equal to 0.05 and a fold change (FC) greater than or equal to 1.4 were further analysed.

Gene annotation was performed based on similarity scores in Basic Local Alignment Search Tool (BLASTN) (National Library of Medicine, USA) comparisons against ovine or bovine sequences in GenBank. Gene lists containing significantly changed genes were submitted to GenBank for annotation based on similarity scores in BLASTN comparisons against bovine and ovine sequences and to Affymetrix Netaffx Analysis Centre^53^ for Bovine Affymetrix® GeneChip® 3′IVT array probe set matches to both the Ovine GeneChip® Gene 1.0 ST Expression Array and mammalian orthologues through searches of gene names, gene symbols, probe set ids, Gene Ontology (GO) and Medical Subject Heading (MeSH) terms, accession identities and probe sequences. These additional gene identifiers were incorporated into the subsequent functional analysis.

#### Pathway analysis

The [networks, functional analyses, etc.] were generated through the use of IPA (QIAGEN Inc., https://www.qiagenbioinformatics.com/products/ingenuity-pathway-analysis)^54^. The Ingenuity® Knowledge Base (http://www.ingenuity.com/science/knowledge-base) contains a database of manually curated and experimentally validated physical, transcriptional and enzymatic molecular interactions and covers primarily human, mouse, and rat genes, however bovine genes are also accepted although the coverage is not as extensive. For IPA analysis, a dataset containing gene identifiers and corresponding expression and P-values were uploaded into IPA. Both the Affymetrix® GeneChip® Bovine Genome Array and sheep gene orthologue accession identities were used as reference gene sets and all genes, called focus genes, with an adjusted p*-* value ≤ 0.05 were included.

Functional analysis of focus genes was performed using IPA to characterise biological functions. For this, IPA performed an over representation analysis that categorises the differentially regulated focus genes within the uploaded lists into functional groups using the Ingenuity® Knowledge Base. Each category in IPA is ranked based on the number of genes falling into each functional group. Significance of relationship to a function or network was assigned by calculating and assessing both the IPA generated P-value and the z-score. The P-value in IPA is estimated by a right-tailed Fisher’s exact test; relationships with *P*-value ≤ 0.05 were considered significant. The z-score algorithm is a statistical measure of the match between the expected relationship direction and the observed gene expression and a score of ≥2 or ≤−2 was considered significant^54^.

Where significant, analysis included the Ingenuity® Knowledge Base enabled integration of focus genes to canonical and predicted pathways, upstream and downstream regulators and predicted non-directional gene interaction networks. In relation to upstream regulator analysis, IPA uses the z-score algorithm to make predictions and to reduce the chance that random data generated the significant predictions and does not take into account the gene expression observed for the predicted upstream regulator itself.

In addition to these bioinformatics tools the Chilibot specialised search software (http://www.chilibot.net/) was utilised to mine the PubMed literature database. This software is designed for identifying relationships between genes, proteins, or any keywords. The Chilibot keyword search algorithm directly presents the key information, i.e. sentences, containing both of the terms, and these sentences are organized into different relationship types based on linguistic analysis of the text. The relationships are summarized into a graph, with links to sentences describing the relationships, as well as the terms themselves^55^.

#### Quantitative PCR verification of microarray results

Quantitative (q)PCR analysis was carried out on selected differentially regulated genes to verify the array findings. RNA samples from all 30 animals within the trial (20 MAP exposed and all 10 control) and from each of the 5 trial time points (2, 10, 18, 32 and 56 weeks post MAP exposure) analysed by microarray (n = 150) were subjected to qPCR analysis. Initially 5 non-changing genes were selected as reference markers from previous studies^37^ as well as from the array dataset; this follows MIQE (Minimum Information for Publication of Quantitative Real-Time PCR Experiments) guidelines^56^. The reference primer suitability was assessed by GeNorm and two were selected for further analysis (Table 1).

**Table 1:** Sequences and predicted specificity of primers used for qPCR validation of array findings.

Gene symbol	Primer sequence (5′-3′)	Ovis Accession	Predicted specificity
STARD10	F-aagagctgcgtcatcacctac	XM_012155230	MAP exposed
	R-gtgtagccacggtttgaagt		
BLA-DQB	F-cagatcaaggttcggtggtt	XM_012173129	MAP exposed
	R-ctctgggcagattcagact		
ITGB3	F-agcagggtgaacccctaaag	XM_012186283	Diseased
	R-ggcatttaggtcaaccagga		
LXN	F-taatagccgtctgccaaagg	XM_004003214.3	Diseased
	R-ggcaccactgctgttgataa		
FCN1	F-cacagttagaggagctctcg	XM_012117952	Diseased
	R-ccgatttgccaagtaccagt		
CES1	F-gacaaggaagtggctttctggaat	XM_004014994	Diseased
	R-acgcctctttggcacataaa		
MYH9 (FST)	F-ggattctctgctgcctgttc	XM_015094855.1	Multibacillary
	R-cctcaccgatgtcttcatca		
DLGAP4	F-actggttcctgaagctgctc	XM_015099858	Multibacillary
	R-ctggaatttctgcgacatca		
BPI	F-aagctgctcctggaactgaa	NM_001306124	Paucibacillary
	R-ggacatctgcaccgaacag		
C10H15orf48	F-gcttcgtcattcgctgtgta	XM_004010648	Resilient
	R-cggaccttctgcaactcttc		
DHRS7	F-atggccaatgatttgaagga	XM_004010693	Resilient
	R-cagtggcttgaaaaatgcaa		
GNG11	F-tcgtttgttgtcgttttcca	NM_001139445	Resilient
	R-gtcatgcacaagtggtgtcc		
TRMT10B	F-gaaggcccaagaacactctg	XM_004004242.3	Resilient
	R-gctgcctccttaggctctct		
Ovine B-Actin	F-catcctgaccctcaagtacc	NM_001009784.2	Reference gene
	R-tcgttgtagaaggtgtggtg		
Ovine GAPDH	F-agaaacctgccaagtatgatg	NM_001190390.1	Reference gene
	R-cctagaatgcccttgagagg		

Thirteen genes of interest were selected for validation of the array data with predicted association specific to disease outcome. Forward and reverse primer pairs were designed for the gene regions of interest using the Primer3 software^57^. The primers were designed to limit amplification of genomic DNA contaminants through adaptation of primer length and selection of sequences specific to the region of interest. Each primer pair was verified to ensure alignment to relevant the *Ovis aries* region of interest using BLASTN. Primers were validated using a serial dilution of cDNA to determine the optimal concentration. Primer sequences used in this study are listed in Table 1 along with the appropriate ovine accession number and the predicted specificity of the gene in relation to the array findings.

qPCR reactions were completed as previously described^58^. Briefly, selected RNA samples (5 µg) were DNase-treated to remove genomic DNA, using 10 µl RQ1 DNAse (Promega) and 1 µl RNasin Plus RNase inhibitor (Promega), then reverse transcribed to cDNA using oligo (dt) and random primers with the AffinityScript qPCR cDNA synthesis kit (Stratagene, Agilent) according to the manufacturers’ instructions. qPCR was performed using an Mx3000P Real-time PCR system (Stratagene, Agilent) using the QuantiTect SYBR Green PCR kit (Qiagen). Assays were prepared in 96 well plates and included duplicates of each sample. Reaction volumes of 25 µl (including 10 µl of target cDNA at a 1/100 dilution) were prepared and amplified under the following conditions: 95 °C for 15 min, then 40 cycles of 95 °C for 20 s, 52–60 °C for 30 s and 72 ^o^C for 30 s, with fluorescence acquisition at the end of each annealing step. The specificity of the reaction was confirmed using melting curve analysis and standard curves were performed on each plate for each primer set. Data collected from the qPCR were analysed using qBASE+ analysis software (Biogazelle) utilising a modified Comparative Ct (∆∆Ct) method^59^.

## Results

### Classification of disease outcome

All samples for transcriptomic and qPCR analysis for this study were collected prior to the onset of clinical signs of paratuberculosis in any animal. The first animal with evidence of weight loss was sacrificed at 56 weeks post exposure to MAP, six were sacrificed between 64 and 66 weeks, and one at 92 weeks. The remainder (n = 12) were sacrificed at 137 weeks (approximately 2 ½ years), with only one of these animals showing evidence of disease at necropsy. Serum antibody ELISA, specific IFN-gamma release assays and faecal cultures were conducted at regular timepoints post-exposure, as previously reported^19^.

Of the MAP exposed sheep, 60% produced faeces with detectable viable MAP at least twice in the trial period (see Begg *et al*. for details^41^). The disease outcome based upon evidence of a positive tissue culture result for MAP-exposed Merino sheep were: diseased 45% (n = 9) and resilient 55% (n = 11). Within the diseased (infected) group, 6 had multibacillary lesions and 3 had paucibacillary lesions. All infected sheep had AFB in tissues and 6 had disseminated infection as viable MAP was detected in liver samples. None of the 11 resilient sheep had demonstrable MAP infection, AFB or histological lesions consistent with MAP in any of the multiple intestinal tissue sections assessed, however most (n = 7) were faecal culture positive (infectious) on one to two occasions (prior to 33 weeks post exposure to MAP) and all had indirect evidence of MAP exposure in the form of specific IFNγ and lymphocyte proliferative responses^19^.

### Microarray differential gene expression

**Figure 1:**
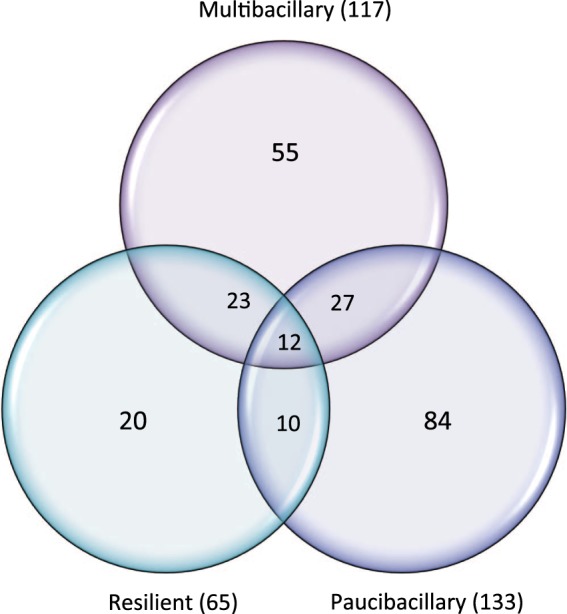
Venn diagram of gene list overlap. The Venn diagram of the distribution of differentially expressed probesets (based on the cut-off criteria p ≤ 0.01 and |FC| ≥ 1.4) between Multibacillary (n = 6), Paucibacillary (n = 3) and Resilient (n = 11) sheep cohorts is presented.

Following statistical analysis of the GeneChip® derived data, genes meeting the criteria for differential expression (FDR ≤0.05 and a FC ≥1.4) were identified consistently across all five sampling times associated with each of the defined groups (paucibacillary, multibacillary or resilient) in comparison to the unexposed control sheep (Fig. 1).

The paucibacillary group of animals returned 133 consistently regulated oligonucleotide probes over time, of which 84 were uniquely expressed within the paucibacillary cohort. These probes are associated to 116 genes, of which 53 were downregulated and 63 were upregulated in comparison to the MAP unexposed controls. Multibacillary animals returned 117 probes (98 genes; 64 downregulated and 34 upregulated) of which 55 probes were uniquely differentially expressed in the multibacillary cohort. The resilient cohort returned 65 significantly regulated probes, 20 of which were unique to this disease classification, associated with 53 genes, (44 downregulated and 9 upregulated). Twelve oligonucleotide probes (11 genes, 8 downregulated and 3 upregulated) were similarly changed in all three cohorts and may be suitable as markers of MAP exposure following validation (Supplementary Table 1, probes identified in bold text and Supplementary Fig. 2 illustrates the kinetics of the genes across the five sampling points). Transcription abundances ranged from 3.4 fold upregulated to −4.2-fold downregulated.

Members of several gene families were regulated, including S100 calcium binding (S100A12, S100A13, S100A8, S100A9), major histocompatibility complex (MHC) class I and class II (Table 2) and associated T cell receptor (T cell receptor beta, delta and gamma); in addition, in paucibacillary animals alone there was downregulated expression of probesets for bacterial cell wall cleaving lysozyme gene product components (lysozyme 1b, lysozyme 3a precursor and lysozyme C-1-like). There is evidence of prolonged overall inhibition of expression of MHC class I genes (LT984574 and XM_015091331) in both the multibacillary and the paucibacillary cohorts. This downregulation is also present in the regulated cohort for the XM_015091331 related probeset Bt.29814.1.S1. Downregulation of MHC class II DQ and DO alpha probesets is present in both the multibacillary and paucibacillary cohorts and in contrast, expression of MHC class II DQ beta gene (XM_012173129) is upregulated in the multibacillary cohort probesets (Bt.23296.1.S1_at, Bt.350.1.S1_at, and Bt.350.1.S1_x_at) and across all three cohorts (Bt.350.1.S1_s_at). Resilient animals show enhanced regulation of MHS class II DQ alpha and beta genes but confoundingly, decreased expression of MHC class II DQ alpha 2, with no probeset overlap.

**Table 2:** MHC Class I and Class II probesets significantly differentially regulated in multibacillary, paucibacillary and resilient cohorts of sheep.

Ovine MHC Class	Bovine Probeset ID	Ovine Gene Symbol	Ovine accession #	Gene Title	Fold Change		
					Multibacillary	Paucibacillary	Resilient
MHC I	Bt.28022.1.A1_s_at	Class I MHC Ovar-N	LT984574	Ovis aries BOLA class I Ovar-N) allele 25:01	−2.7	−1.7	
	Bt.29824.1.S1_s_at	Class I MHC Ovar-N	LT984574	Ovis aries BOLA class I Ovar-N) allele 25:01	−4.2	−3.1	
	Bt.22767.1.S1_at	LOC101104866	XM_015091331	Ovis aries musimon BOLA class I antigen	−2.0	−2.1	
	Bt.29814.1.S1_at	LOC101104866	XM_015091331	Ovis aries musimon BOLA class I antigen	−2.4	−3.0	−2.0
	Bt.5324.1.S1_s_at	LOC101104866	XM_015091331	Ovis aries musimon BOLA class I antigen	−1.4		
	Bt.4762.1.S1_at	LOC101113217	XM_015105290	Ovis aries BOLA class I alpha chain BL3-7-like	1.6	1.5	
	Bt.3805.1.S1_at	LOC105612701	XM_012163337	Ovis aries musimon BOLA class I alpha chain BL3-7	1.6	1.4	
MHC II	Bt.4751.2.S1_a_at	MHC Ovar DQA2	M93431	Ovis aries Merino class II DQ alpha 2.2		−1.7	
	Bt.22867.1.S1_x_at	LOC101108696	NM_001159759	Ovis aries boLa class II DQ alpha			1.8
	Bt.22867.2.A1_at	MHC Ovar DQA2	NM_001308598	Ovis aries HLA class II DQ alpha 2	−2.1		−2.9
	Bt.13510.1.S1_at	LOC101109219	XM_012112137	Ovis aries musimon class II DO beta chain	−1.5		
	Bt.350.1.S1_at	MHC-DQB2	XM_012173129	Ovis aries musimon boLa class II DQB*0101 beta	1.5		
	Bt.350.1.S1_s_at	MHC-DQB2	XM_012173129	Ovis aries musimon boLa class II DQB*0101 beta	3.1	3.4	2.9
	Bt.350.1.S1_x_at	MHC-DQB2	XM_012173129	Ovis aries musimon boLa class II DQB*0101 beta	1.5		

The significantly regulated probesets and related genes are shown in Supplementary Table 1 with associated ovine accession number and Ovine gene title. The raw microarray data have been deposited in the National Centre for Biotechnology Information’s Gene Expression Omnibus (GEO) and are accessible through GEO Series accession number GSE114384.

### Pathway analysis

Not all of the genes identified as differentially regulated in the preliminary analysis were successfully mapped to a molecule or biological pathway within the Ingenuity® Knowledge Base. Among the mapped focus genes, the following were network eligible: paucibacillary 92, multibacillary 80 and resilient 40 including similarly changed genes across all three cohorts (Supplementary Tables 3–5).

The gene lists were interrogated based on molecular or cellular functional annotation to assess if a functional gene category contained an over-representation of genes relative to the microarray reference gene list. The top ten functional categories returned for each cohort are listed in Table 3, highlighting significant z-scores, p-values and predicted activation states of the defined category. Since none of the paucibacillary focus gene-associated functional categories returned a significant z-score, Table 3 includes paucibacillary disease associated functional categories with significant p-values. Common to all outcome variables were differential over-representation of expression of genes related to *cellular growth and proliferation* and *cell movement*, with a predicted overall decrease in activation. Also common between all three groups were over-representation of focus genes aligned to *lipid metabolism* functions, although there was variation of the gene profiles and the predicted activation states between cohorts (Table 4). Upstream regulator analysis identified molecules including cytokines, transcription regulators and kinases that may be responsible for the gene expression changes observed in the experimental dataset (Table 5).

**Table 3:** Top ten molecular or biological functional annotations.

Disease cohort	Categories	Diseases or Functions Annotation	p-Value	Predicted Activation State	Activation z-score	Molecules	#
Multibacillary	*Cellular Growth and Proliferation*	proliferation of cells	5.32E-04	Decreased	−2.9	*BASP1,CSNK1G3,CYBB,CYP4F2,FABP5,FGL2,FST,GPNMB,IGF2BP3,JUN,KLF5,LY86,MAGI1,MARCKS,MXD1,MXI1,PDK4,RARRES1,RSPO1,S100A13,S100A8,S100A9,STARD10,TBC1D8,TGM1,TJP1,TNFAIP6,TNFSF13B,TNS3,UCHL1*	30
		proliferation of endothelial cells	1.52E-02	Decreased	−2.2	*CYBB,CYP4F2,JUN,S100A8,S100A9*	5
	*Cellular Movement*	invasion of cells	1.02E-03		−1.9	*FABP5,FST,IFIT2,IGF2BP3,JUN,MAGI1,MARCKS,PDLIM1,S100A12,S100A8,S100A9,TJP1*	12
	*Lipid Metabolism*	concentration of lipid	5.47E-04	Decreased	−2.2	*C1QA,CES1,CYBB,CYP4F2,FABP5,JUN,P2RY13,PDK4,S100A12,S100A8,S100A9,STARD10*	12
		concentration of fatty acid	1.04E-02	Decreased	−2.2	*CES1,CYP4F2,P2RY13,PDK4,STARD10*	5
	*Organismal Development*	angiogenesis	3.65E-02	Decreased	−2.9	*C1QA,CYBB,CYP4F2,JUN,KLF5,S100A12,S100A8,S100A9,TJP1*	9
	*Organismal Survival*	survival of organism	7.38E-03		1.9	*CYBB,DNAJC5,FGL2,FST,GCA,KLF5,MAGI1,S100A9,TNFSF13B*	9
	*Tissue Morphology*	growth of epithelial tissue	5.25E-03	Decreased	−2.8	*CYBB,CYP4F2,FST,JUN,KLF5,S100A8,S100A9,TGM1,TNFAIP6*	9
		quantity of connective tissue	2.50E-02	Increased	2.2	*CES1,FABP5,GPNMB,JUN,MXD1,P2RY13,PDK4*	7
		growth of connective tissue	4.07E-02	Decreased	−2.4	*FST,JUN,KLF5,S100A9,STARD10,TGM1,TNFSF13B*	7
Paucibacillary	*Cellular Growth and Proliferation*	proliferation of cells	2.60E-02		−1.5	*AKR1C3,BPI,CD14,CEBPD,CTNNA1,DCBLD2,DTX1,FABP3,HSPB8,IGF2BP3,ITGB3,KCTD12,LY86,MTA3,MYH11,NAGA,NPPC,PAWR,PDK4,RARRES1,RSPO1,SLC39A12,STARD10,TES,TJP1,TNS3,TRIB2,ZBTB32*	28
	*Cellular Movement*	cell movement	1.82E-02		−1.0	*CAPG,CD14,CEBPD,CES1,COL11A1,CTNNA1,CXCR6,CXXC5,DCBLD2,DTX1,IGF2BP3,ITGB3,MYH11,PLA2G7,PPIC,RARRES1,TJP1,TNS3,TSPAN7*	19
		migration of cells	4.62E-02		−1.0	*CAPG,CD14,CES1,COL11A1,CTNNA1,CXXC5,DCBLD2,DTX1,IGF2BP3,ITGB3,MYH11,PLA2G7,PPIC,RARRES1,TJP1,TNS3*	16
	*Metabolic Disease*	glucose metabolism disorder	1.08E-02			*AKR1C3,BPI,CAPG,CEBPD,CLIC5,COL11A1,CXCR6,FABP3,ITGB3,LY86,MYH11,PARD3B,PLD4*	13
	*Endocrine System Disorders*	diabetes mellitus	4.72E-03			*AKR1C3,BPI,CAPG,CEBPD,CLIC5,COL11A1,CXCR6,ITGB3,LY86,MYH11,PARD3B,PLD4*	12
	*Tissue Morphology*	morphology of connective tissue cells	3.72E-03			*CEBPD,CES1,CXXC5,DCBLD2,ITGB3,PDK4*	6
	*Infectious Diseases*	Bacterial Infections	1.73E-02		−0.2	*BPI,CAPG,CD14,FCN1,LY86,PLA2G7*	6
	*Cellular Development*	differentiation of hematopoietic progenitor cells	1.90E-02			*CEBPD,DCBLD2,DTX1,MYH11,PLD4*	5
	*Molecular Transport*	concentration of D-glucose	2.84E-02		0.0	*CES1,FABP3,ITGB3,NPPC,PDK4*	5
	*Lipid Metabolism*	transport of lipid	2.94E-02		−0.1	*CD14,CES1,FABP3,ITGB3*	4
Resilient	*Cellular Growth and Proliferation*	proliferation of cells	5.64E-05	Decreased	−2.0	*A2M,ACSL6,CSNK1G3,CYBB,EPB41L3,FABP3,GPNMB,HNF4A,KLF5,MAP2,NNAT,PAWR,PTGS2,RSPO1,S100A9,SLC39A12,STARD10,THBS1,TIMP2,TJP1,TNFSF13B,TNS3,VCAN*	23
		proliferation of smooth muscle cells	9.92E-03		−2.0	*KLF5,PTGS2,THBS1,VCAN*	4
	*Cellular Movement*	migration of cells	9.23E-04		−1.9	*A2M,CYBB,GPNMB,KLF5,MAP2,OLR1,PTGS2,S100A9,THBS1,TIMP2,TJP1,TNFSF13B,TNS3,VCAN*	14
		cell movement of leukocytes	8.75E-03		−1.8	*CYBB,PTGS2,S100A9,THBS1,TIMP2,TNFSF13B,VCAN*	7
		cell movement of smooth muscle cells	1.52E-03		−1.9	*A2M,PTGS2,THBS1,VCAN*	4
		cell movement of macrophages	8.26E-03		−1.9	*CYBB,PTGS2,THBS1,VCAN*	4
	*Lipid Metabolism*	concentration of lipid	3.52E-03		−1.9	*CYBB,FABP3,HNF4A,OLR1,P2RY13,PTGS2,S100A9,STARD10*	8
		concentration of cholesterol	1.58E-04		−1.7	*CYBB,HNF4A,OLR1,P2RY13,PTGS2,STARD10*	6
	*Molecular Transport*	secretion of molecule	5.47E-03	Decreased	−2.2	*A2M,OLR1,PTGS2,RSPO1,S100A9,THBS1*	6
	*Organismal Survival*	organismal death	1.72E-03		1.9	*A2M,CYBB,ELAVL3,EPB41L3,FABP3,GPNMB,HNF4A,KLF5,MAP2,PTGS2,RSPO1,S100A9,THBS1,TIMP2,TNFSF13B,VCAN*	16

**Table 4:** Functional annotation within the category of *lipid metabolism*.

	Diseases or Functions Annotation	p-value	z-score	Molecules	# Molecules
**Multibacillary**	*concentration of lipid*	5.5E-04	−2.2	*C1QA,CES1,CYBB,CYP4F2,FABP5,JUN,P2RY13,PDK4,S100A12,S100A8,S100A9,STARD10*	12
	*concentration of fatty acid*	1.0E-02	−2.2	*CES1,CYP4F2,P2RY13,PDK4,STARD10*	5
	*concentration of triacylglycerol*	5.2E-04	−0.9	*C1QA,CES1,CYBB,FABP5,JUN,PDK4,STARD10*	7
	*concentration of cholesterol*	1.8E-03	−0.7	*CES1,CYBB,FABP5,JUN,P2RY13,STARD10*	6
	*fatty acid metabolism*	7.1E-03	0.5	*CES1,CYP4F2,FABP5,KLF5,PDK4,S100A12,S100A8,S100A9*	8
	*efflux of cholesterol*	1.1E-03	0.9	*CES1,S100A12,S100A8,S100A9*	4
	*transport of lipid*	3.6E-03	1.5	*CES1,FABP5,S100A12,S100A8,S100A9*	5
**Paucibacillary**	*transport of lipid*	2.9E-02	−0.1	*CD14,CES1,FABP3,ITGB3*	4
	*catabolism of lipid*	1.7E-02		*AKR1C3,CES1,PLA2G7*	3
	*binding of lipopolysaccharide*	3.0E-03		*BPI,CD14*	2
	*transmission of lipid*	5.4E-03		*CD14,CES1*	2
	*hydrolysis of triacylglycerol*	1.2E-02		*FABP3,PLA2G7*	2
	*oxidation of palmitic acid*	2.2E-02		*FABP3,PDK4*	2
	*quantity of diacylglycerol*	2.5E-02		*CES1,PDK4*	2
	*transport of fatty acid*	4.0E-02		*CES1,FABP3*	2
**Resilient**	*concentration of lipid*	3.5E-03	−1.9	*CYBB,FABP3,HNF4A,OLR1,P2RY13,PTGS2,S100A9,STARD10*	8
	*concentration of cholesterol*	1.6E-04	−1.7	*CYBB,HNF4A,OLR1,P2RY13,PTGS2,STARD10*	6
	*synthesis of eicosanoid*	3.2E-03	−1.2	*HNF4A,KLF5,OLR1,PTGS2*	4
	*fatty acid metabolism*	1.9E-03	−1.0	*ACSL6,FABP3,HNF4A,KLF5,OLR1,PTGS2,S100A9*	7
	*concentration of fatty acid*	1.5E-03	−0.5	*FABP3,OLR1,P2RY13,PTGS2,STARD10*	5
	*uptake of lipid*	1.1E-03	0.0	*A2M,FABP3,OLR1,THBS1*	4
	*uptake of long chain fatty acid*	3.1E-05		*FABP3,OLR1,THBS1*	3
	*homeostasis of phospholipid*	2.7E-04		*FABP3,HNF4A*	2
	*uptake of palmitic acid*	9.8E-04		*FABP3,OLR1*	2
	*homeostasis of lipid*	1.4E-03		*FABP3,HNF4A,PTGS2,STARD10*	4
	*transport of long chain fatty acid*	2.5E-03		*ACSL6,FABP3*	2

**Table 5:** Upstream regulator analysis identified molecules.

	Upstream Regulator	Molecule Type	Predicted Activation State	z-score	Target molecules in dataset
Multibacillary	IFNG	cytokine	Inhibited	−3.6	*C1QA,CYBB,FABP5,FGL2,HLA-B,HLA-DOB,IFIT2,JUN,P2RY14,RARRES1,S100A8, S100A9,TGM1,TJP1,TNFAIP6,TNFSF13B*
	IL1B	cytokine	Inhibited	−2.6	*CYBB,FABP5,FST,JUN,S100A8,S100A9, TGM1,TJP1,TNFAIP6,TNFSF13B*
	OSM	cytokine	Inhibited	−2.4	*GCA,GPNMB,HLA-B,JUN,MARCKS,RAB31,S100A12,S100A8,S100A9*
	Interferon alpha	group	Inhibited	−2.2	*HLA-B,IFIT2,S100A9,TGM1,TNFSF13B*
	TGFB1	growth factor	Inhibited	−2.1	*C1QA,CXCR6,CYBB,FABP5,FILIP1L,JUN,MXI1,P2RY14,RAB31,TNFAIP6,TNFSF13B*
	TGM2	enzyme	Inhibited	−2.0	*GCA,HLA-B,IFIT2,S100A8*
	CREB1	transcription regulator	Inhibited	−2.0	*CRYM,FGL2,GPNMB,JUN,KLF5*
	PRKCA	kinase	Inhibited	−2.0	*CYBB,IGF2BP3,JUN,S100A8,S100A9*
	IL1A	cytokine	Inhibited	−2.0	*JUN,S100A12,S100A8,S100A9*
	IGF1	growth factor	Inhibited	−2.0	*C1QA,CYBB,JUN,TJP1*
	TNF	cytokine	Inhibited	−2.0	*CYBB,FABP5,FST,HLA-B,JUN,KLF5,MAGI1,S100A8,S100A9,TBC1D8,TJP1,TNFAIP6,TNFSF13B,TNS3*
	HOXA10	transcription regulator	Activated	2.0	*CYBB,FST,S100A12,TNFSF13B*
	Immunoglobulin	complex	Activated	2.0	*RAB31,S100A12,S100A8,S100A9*
Paucibacillary	NEUROG1	transcription regulator	Inhibited	−2.0	*FABP3,ITGB3,PPIC,TSPAN7*
	TP53	transcription regulator		2.0	*ACTA2,CLU,CTSH,DTX1,FABP3,HLA-B,ITGB5,PAWR,PLA2G16,PPIC,PSMD8,SH3BGRL2,TJP1,VCAN*
	PGR	ligand-dependent nuclear receptor		2.0	*AKR1C3,CEBPD,GSTM3,VCAN*
	EDN1	cytokine		2.0	*ACTA2,ITGB3,OLR1,VCAN*
	BRD4	kinase		2.0	*ACTA2,HBD,ITGB5,PDGFC*
	CEBPB	transcription regulator		2.0	*ACTA2,AKR1C3,CD14,CEBPD,CFD,PDK4,VCAN*
	CXCL12	cytokine	Activated	2.2	*ACTA2,AKR1C3,CD14,CXCR6,ITGB3*
	RAF1	kinase	Activated	2.2	*CTSH,DCBLD2,ITGB3,ITGB5,SELENBP1*
	ERK	group	Activated	2.2	*CEBPD,CLU,ITGB3,PDGFC,PDK4,VCAN*
	AKT1	kinase	Activated	2.2	*ACTA2,CEBPD,CLU,MYH11,VCAN*
	APP	other	Activated	2.6	*ACTA2,CLU,CRYL1,FABP3,GSTM3,ITGB3,MAP2,OLR1,SHISA3,TJP1,VCAN*
Resilient	IL1B	cytokine	Inhibited	−3.1	*A2M,CYBB,HNF4A,OLR1,PTGS2,S100A9,THBS1,TIMP2,TJP1,TNFSF13B,VCAN*
	IFNG	cytokine	Inhibited	−2.6	*A2M,CYBB,PTGS2,S100A9,THBS1,TJP1,TNFSF13B*
	TNF	cytokine	Inhibited	−2.4	*A2M,CYBB,HNF4A,KLF5,OLR1,PTGS2,S100A9,THBS1,TIMP2,TJP1,TNFSF13B,TNS3*
	CREB1	transcription regulator	Inhibited	−2.2	*CRYM,GPNMB,KLF5,NNAT,PTGS2*
	P38 MAPK	group	Inhibited	−2.2	*CYBB,KLF5,PTGS2,THBS1,TIMP2*
	TGFB1	growth factor	Inhibited	−2.2	*CYBB,HNF4A,OLR1,PTGS2,THBS1,TIMP2,TNFSF13B,VCAN*
	EDN1	cytokine	Inhibited	−2.2	*CYBB,OLR1,PTGS2,THBS1,VCAN*
	PI3K (complex)	complex		−2.0	*CYBB,MAP2,PTGS2,THBS1*
	ERK	group		−2.0	*KLF5,PTGS2,THBS1,VCAN*
	PPARA	ligand-dependent nuclear receptor		−2.0	*CYBB,FABP3,HNF4A,PTGS2,TJP1*

### Validation of array results by qPCR

**Figure 2:**
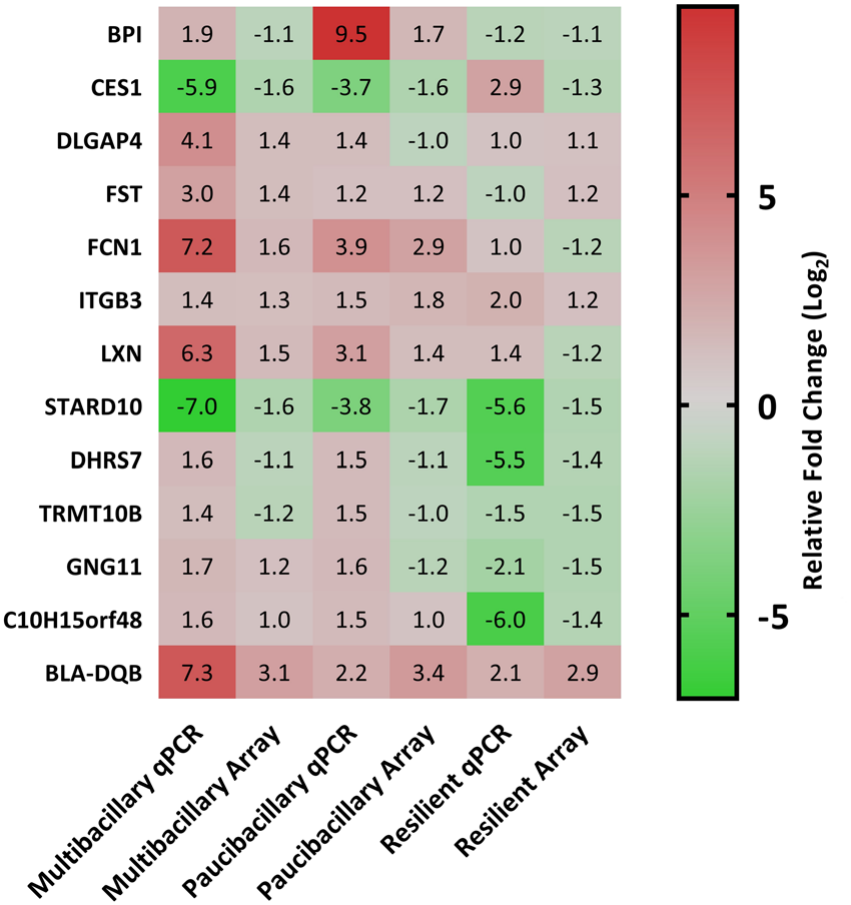
Comparison of expression levels measured with array hybridization and qPCR. Log_2_ transformed fold changes in RNA levels (MAP treated variables/control) were plotted for both array and qPCR. Fold changes in RNA levels measured by microarray and qPCR showed good correlation (Pearson’s correlation coefficient, r = 0.91). Explanation of abbreviations of gene names are provided in the Supplementary Table 1.

Thirteen genes differentially regulated in the array analysis were selected for validation by qPCR of 150 trial derived samples from all trial animals (n = 20 MAP exposed and n = 10 unexposed controls) across the time course of disease (2–56 weeks) using *Ovis aries* mapped primers. There was good correlation between the array and qPCR (Pearson’s correlation coefficient r− = 0.91). The annotated heatmap (Fig. 2) illustrates consistency in the direction of changes in fold change in the expression of the MAP-exposed cohort variables (multibacillary, paucibacillary or resilient in comparison to the control) for all genes relevant to each variable (Supplementary Tables 1 and 2), confirming validity of array results.

## Discussion

Paratuberculosis remains an important livestock disease despite implementation of management programmes. Lack of progress in eradicating the disease from livestock is due in part to limitations inherent in diagnostic tests. These cannot reliably identify subclinically infected, infectious animals, leading to on-going spread of the infection. Research on this aspect is needed to develop better technologies but has been hampered by lack of consistency in classification of MAP exposed animals^40^ in studies aimed at developing an understanding of pathogenesis mechanisms and the relationship between MAP and its host.

This study reports array-derived transcriptomic changes in the early subclinical phase of ovine paratuberculosis. Blood samples for RNA analysis were collected from MAP exposed animals, prior to detection of clinical disease, and animals were retrospectively classified into cohorts (multibacillary, paucibacillary and resilient) using detailed microbiological and pathological analysis. This design has enabled an in-depth analysis of temporally consistent gene expression changes following MAP exposure, with emphasis on the early subclinical phase of the disease. Age matched controls held under the same environmental conditions were sampled in parallel and the timeframe of collection spanned an entire year, thus including all seasonal conditions. Modulation of the host transcriptome in response to MAP infection was evident from the number of genes differentiating between the three outcomes (multibacillary, paucibacillary and resilient). A key outcome was that the genes identified were consistently modulated across the disease timecourse, from early in the infection (2 weeks post-exposure) until just prior to the first presentation of clinical disease (56 weeks post-exposure). This enabled the identification of putative biomarkers for diagnostic use; a selection of these were validated by qPCR however further validation of these putative biomarker gene panels within naturally infected sheep is warranted, to ensure specificity to MAP infection and disease outcome. These could incorporate pathogen-specific responses such as the MAP-specific IFNγ response and faecal shedding of the pathogen, as we have previously identified^35^.

Interpretation of the differentially regulated genes into molecular and functional categories provides insight into the immunobiology of MAP exposed animals. However, no single database is capable of elucidating the complete picture to tease out all possible interactions^60^ especially when taking into consideration variations inherent between species, adaptation of gene names and acceptable accession numbers.

We previously reported on a small scale transcriptomic study identifying gene expression in response to early MAP exposure in experimentally exposed Holstein cattle during the subclinical phase of paratuberculosis^58^. The MAP exposed cattle were selected from the trial cohort based upon a higher IFNγ response at 4 months post exposure; we hypothesised that this cohort was potentially less susceptible to development of disease if the mechanism of pathogenesis in cattle is similar to that of sheep. Of particular interest was the finding that, following exposure to MAP, the host immune response appears to be driven to enhance the expression of genes related to the ability of cells to present antigenic peptides to CD8^+^ T lymphocytes rather than the CD4^+^ pathway that is commonly associated with T helper lymphocyte responses. In the present study, the MHC genes were amongst the most abundantly changed groups of common genes in the Merino sheep with evidence of either paucibacillary or multibacillary disease. In particular, oligonucleotide probes associated with both MHC class I and class II complex alleles or domains were regulated (Table 2). The genes exhibiting these domains encode for highly modular proteins in which the variable and constant domains have clear, conserved sequence patterns^61,62^. The MHC is a vital component of the immune milieu; mature T cells are activated when the TCR-CD3 complex on the surface of cells encounter antigens associated with MHC on antigen presenting cells^63,64^. The mechanism of activation requires a sustained interaction with the antigen-MHC complex to proliferate and initiate downstream signalling responses^65^. Overall this suggests that multibacillary animals will express a propensity related to the ability of cells to present antigenic peptides to CD4^+^ T lymphocytes rather than the CD8^+^ pathway; this would agree with the commonly associated T helper lymphocyte responses. Paucibacillary sheep show a similar trend but with fewer supporting probesets, while resilient animals show a response more weighted to MHC class II expression. Of interest is the finding of differential regulation of TCR gene-related probesets. Each TCR is a dimer consisting of one alpha and one beta chain or one delta and one gamma chain. Multibacillary animals show an upregulation of TCR gamma and delta probesets. Paucibacillary animals show upregulation of a delta chain gene probeset but downregulation of a beta chain gene probeset. Resilient animals show enhanced regulation across both delta and beta gene probesets.

It is not known at this stage, whether the *in vivo* alteration to the MHC gene profile is driven by the pathogen or by the host nor what effect this alteration has on the long-term outcome for the pathogenesis of paratuberculosis in sheep. However, any alteration of the expression of MHC genes following exposure to MAP may have a significant impact on the ability of the animal to respond to infection. An explanation for this finding may be informed by the responses of human and mouse cell lines to *Mycobacterium tuberculosis* (*M. tuberculosis*) which modulates host MHC expression to evade detection^66^. This mycobacterium inhibits the expression of MHC class II by human peripheral blood monocytes^67^. Macrophages infected with *M. tuberculosis* exhibit decreased MHC class II and subsequent decreased antigen presentation, resulting in reduced CD4^+^ T cell recognition of infected macrophages^68^. Further investigation of this mechanism led to the identification of lipoproteins as the mycobacterial component that inhibits the MHC via a Toll-like receptor-mediated pathway^69,70^. Very little is known regarding potential MHC allele-based susceptibility in ovine paratuberculosis in relation to multibacillary or paucibacillary disease, although a study carried out on Merino sheep exposed to MAP identified MHC alleles with possible associations with susceptibility or resistance^71^. Verschoor *et al*. identified modulation of the MHC class II alpha precursor in adult Holstein and Jersey cows with subclinical Johne’s disease^72^ and David *et al*. identified modulation of MHC genes in a transcriptomic analysis of experimentally infected Holstein-Friesian calves^30,31,73^, which is supported by our previous finding of very early differential regulation of MHC genes in young exposed cattle. We propose that these genes are consistently regulated and that these early changes may persist in the on-going subclinical infection within the adult host, although to a much lesser degree.

In the host, MAP preferentially resides in macrophages^74^ and activation of MHC class II molecules within this cell type is induced by IFNγ, which signals through its receptor to activate transcription (JAK–STAT) signalling, resulting in STAT1a phosphorylation^75^. This mechanism results in the induction of genes, including the gene encoding a MHC class II transactivator (CIITA) which accumulates with other transcription regulators to bind to the promoters of MHC class II genes and other genes related to antigen processing^76^. The resulting MHC class II molecules participate in antigen processing and presentation^77,78^. Within this study IFNγ was identified as an upstream regulator of sixteen genes within the multibacillary cohort (Table 5) including MHC and transcription regulators. Further transcription regulators and G-protein coupled receptors were regulated within the multibacillary animals (KLF5, MXD1, BASP1, MXI1, JUN, FOS, ZSCAN26, PDLIM1, IGF2BP3, CXCR6) and functional analysis of the genes suggested enhanced *activation of Th2 pathways* but inhibition of *B cell lymphocyte differentiation, Th1 cell activation, migration of cells* and *cell movement*. Of particular interest was the consistent regulation of the JUN and FOS genes since homologs of these constitute the Activator Protein 1 (AP-1) complex and are involved in many cellular events including cellular proliferation and in recent literature AP-1 complex proteins have been implicated in mediation of host phagosomal maturation in defence against *M. tuberculosis*^79^. The process of phagosomal maturation is fundamental in the development of both innate and adaptive immune responses^80^. Over forty years ago the capacity for *M. tuberculosis* to inhibit phagosome-lysosome fusion was first reported^81^ and since then extensive research has illuminated the capacity for *M. tuberculosis* and other mycobacteria to arrest phagosomal maturation and exploit this organelle as a site of replication^82^. Consistent inhibition of AP-1 constituents and related genes (TJP1, JUN, S100A8) in the multibacillary cohort may be related to the host mycobacterial immune evasion strategy and exploratory analysis suggests a connection between IFNγ mediated regulation of AP-1 associated target molecules. This association was not evident within the paucibacillary animals that instead showed consistent modulation of several different transcription regulators (ZBTB32, DTX1, PAWR, SEPT10, CEBPD) and the G-protein coupled receptor (CXCR6). These are predicted to be associated with enhanced *cellular proliferation of T lymphocytes*, *B lymphocytes* and *enhanced functions of leukocytes*. There is evidently a marked variation between differentially expressed transcription regulators in the two disease cohorts. The resilient animals shared similarity with the multibacillary animals for one transcription regulator (KLF5) but apart from that, none of the transcription genes (OLR1, P2RY13, PAWR, HNF4A) or other associated genes are functionally linked to MHC or to T or B lymphocyte functions.

**Figure 3:**
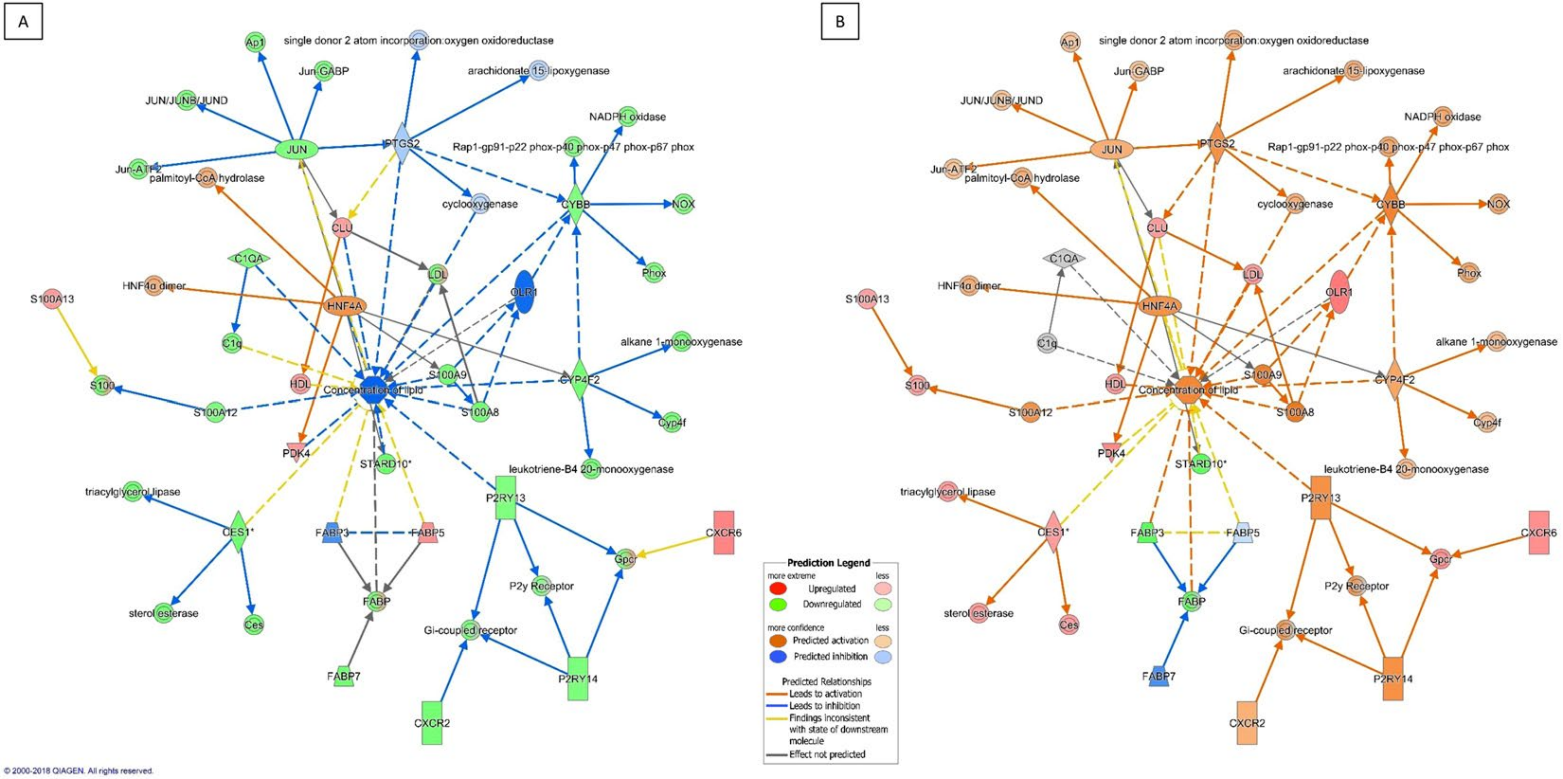
Visual representation of consistently regulated differentially expressed genes in Multibacillary (**A**) and Paucibacillary (**B**) sheep compared to Control sheep. The network maps illustrate lipid concentration associated gene interaction and associated molecular functions/genes with enhanced/upregulated expression are coloured red and those with decreased/downregulated expression in comparison to MAP unexposed control sheep are coloured green. The relationships between the genes and functions are indicated by the colour of the dotted lines as defined in the associated legend. Overall predicted activation of function in response to the gene expression is denoted with orange whereas blue indicates inhibition of function. The networks analyses were generated through the use of IPA (QIAGEN Inc., https://www.qiagenbioinformatics.com/products/ingenuity-pathway-analysis)^54^.

As essential components of the plasma membrane of eukaryotic cells, lipids and in particular cholesterol play an important role in the phagocytosis of pathogenic mycobacteria by macrophages^83–85^ but conversely are utilised by the mycobacteria in infectivity and virulence mechanisms^86,87^. Lipids act as an energy source for *M. tuberculosis*^88,89^ and mycobacteria require lipid support for ongoing intracellular survival. We have previously reported evidence supporting differential regulation of lipid genes in experimentally exposed cattle^58^ and further, we have identified a putative mechanistic pathway of lipid and cholesterol metabolism genes in MAP infection in cattle^90^. The overrepresentation of genes involved in *lipid metabolism* (Table 4) was thus not an unexpected finding. We further identified variations in lipid mechanisms between the multibacillary and the paucibacillary cohorts. Multibacillary sheep consistently expressed genes functionally related to decreased *concentration of lipids* (Fig. 3A)*, cholesterol and fatty acids* (C1QA, CES1, CYBB, CYP4F2, FAB5, JUN, P2RY13, PDK4, S100A12, S100A8, S100A9, and STARD10). It is interesting to note that the lipoprotein binding pattern recognition receptor, complement component 1qA (C1QA, multibacillary fold change -1.5) has been shown to modulate the expression of clusters of genes involved in cell death and apoptosis and in particular this gene promotes macrophage survival in the presence of cholesterol and improves macrophage foam cell function^91^. The predicted suppression of this and other lipid associated genes as illustrated in Fig. 3(A), suggested that the consistent modulation of genes in the multibacillary cohort may be an attempted host-driven, protective mechanism. This is further supported by the finding that accumulation of cholesterol within macrophages impairs phagosomal maturation^92^ thus gene expression that results in inhibition of the *concentration of lipids* and *cholesterol* as seen in both multibacillary and resilient animals (Supplementary Fig. F1) may encourage phagosomal maturation which, in the case of the multibacillary animals, may be further enhanced by the consistent downregulation of JUN, and other AP-1 related genes. The Merino sheep classified as paucibacillary consistently enhanced expression of genes PLA2G7, CES1, and AKR1C3 whose expression is functionally related to the activation of c*atabolism of lipids*. Similarly, the expression of the genes FABP3, CD14, CES1, and ITGB3 predict suppression of the *transport and transmission of lipids and the transport of fatty acid*. This suggests that for paucibacillary animals there may be increased concentration of lipids, supported by the finding that the expression of the genes STARD10 and FABP3 were supressed and IPA predicts that this will lead to enhanced *concentration of lipids* (Fig. 3B). Further molecular analysis is warranted, since it is not possible to definitively state disease immunopathogenesis mechanisms based upon gene expression alone. However, evidence of temporally consistent gene expression changes throughout the subclinical phase of infection in sheep suggests that lipid functions are integral to pathogenesis.

Potentially related to the lipid function is the downregulation in the paucibacillary cohort of probesets for lysosome genes. Lysozyme is an enzyme that cleaves peptidoglycan in bacterial cell walls by catalysing the hydrolysis of (1,4) linkages between the Nacetylglucosamine (NAG) and N-acetylmuramic acid saccharides^93^. Both *M. tuberculosis* and *M. smegmatis* exhibit defence strategies against the lytic activity of lysozyme through expression of surface lipoproteins^94^ and the decreased expression of lysozyme genes in the paucibacillary cohort of sheep suggests existence of a similar mechanism.

The results identify molecular interactions occurring within sheep exposed to MAP, knowledge that may shed light on pathogenesis and suggest genes that were identified as predictive of disease outcome and may be susceptibility factors or potential targets for diagnosis, breeding decisions, vaccination and therapeutics. This study has identified important functional pathways that help explain the differences between paucibacillary and multibacillary disease and the resilient cohort of sheep. These findings strongly support the hypothesis that multibacillary and paucibacillary disease phenotypes develop along related yet distinct pathogenesis pathways. Further, there is evidence of significant variations in the gene expression and associated molecular pathways in resilient animals which appear to have evaded or successfully contained MAP infection.

## Supplementary information

Dataset 1

## Data Availability

The raw microarray data have been deposited in the National Centre for Biotechnology Information’s Gene Expression Omnibus (GEO) and are accessible through GEO Series accession number GSE114384.
