# Supplementary material for: Gene expression profiles during subclinical Mycobacterium avium subspecies paratuberculosis infection in sheep can predict disease outcome
Source: Sci Rep. 2019 Jun 3;9:8245. doi: 10.1038/s41598-019-44670-w (PMC6547741; doi:10.1038/s41598-019-44670-w)
Supplement: Supplementary file 1 — Dataset 1 [file 41598_2019_44670_MOESM1_ESM.docx]

**Gene expression profiles during subclinical *Mycobacterium avium* subspecies *paratuberculosis* infection in sheep can predict disease outcome**

^1^Auriol C Purdie*, ^1^Karren M. Plain, ^1^Douglas J. Begg, ^1^Kumudika de Silva, ^1^Richard J. Whittington

^1^Farm Animal Health, Sydney School of Veterinary Science, Faculty of Science, University of Sydney

*Corresponding author:

Mailing address: University of Sydney, Faculty of Veterinary Science, Private Bag 4003, Narellan, NSW 2567, Australia

Tel: +61 2 90367743

Fax: +61 2 93511618

e-mail: auriol.purdie@sydney.edu.au


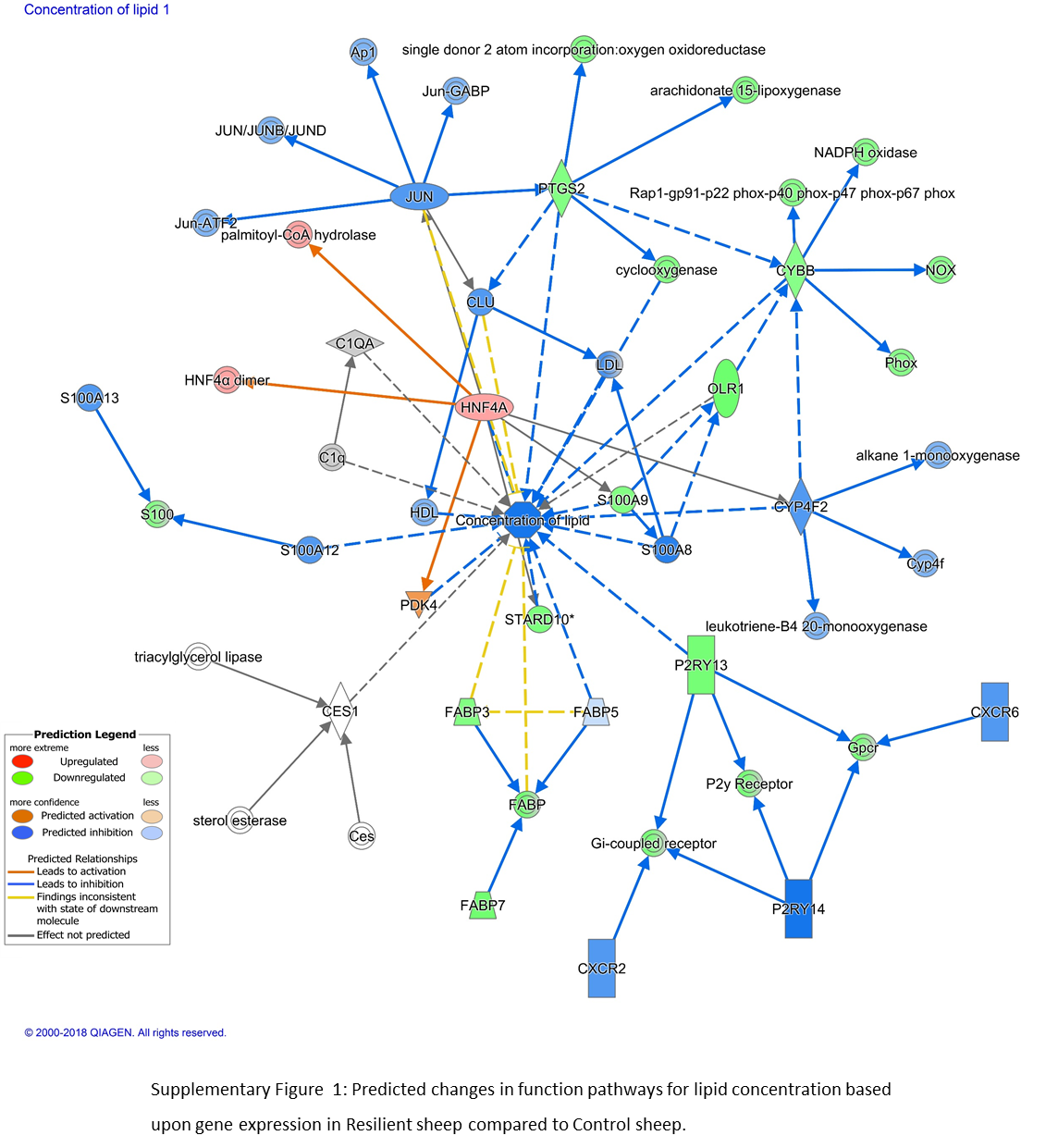


Supplementary Figure 1: Visual representation of consistently regulated differentially expressed genes in Resilient sheep compared to Control sheep. The network maps illustrate lipid concentration associated gene interaction and associated molecular functions/genes with enhanced/upregulated expression are coloured red and those with decreased/downregulated expression in comparison to MAP unexposed control sheep are coloured green. The relationships between the genes and functions are indicated by the colour of the dotted lines as defined in the associated legend. Overall predicted activation of function in response to the gene expression is denoted with orange whereas blue indicates inhibition of function. The networks analyses were generated through the use of IPA (QIAGEN Inc., https://www.qiagenbioinformatics.com/products/ingenuity-pathway-analysis)^54^.


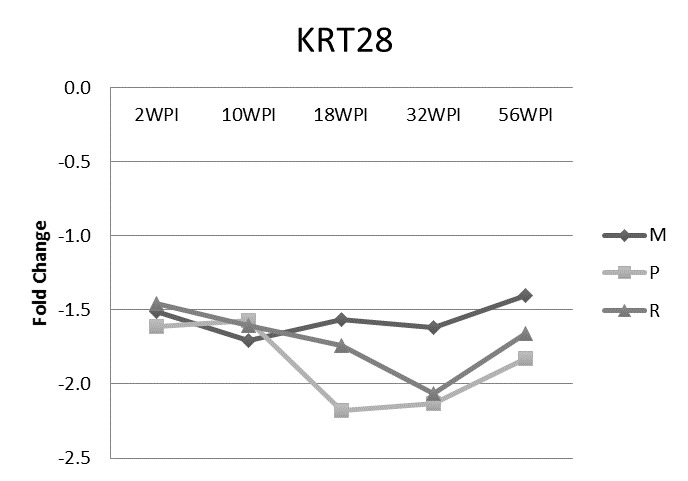

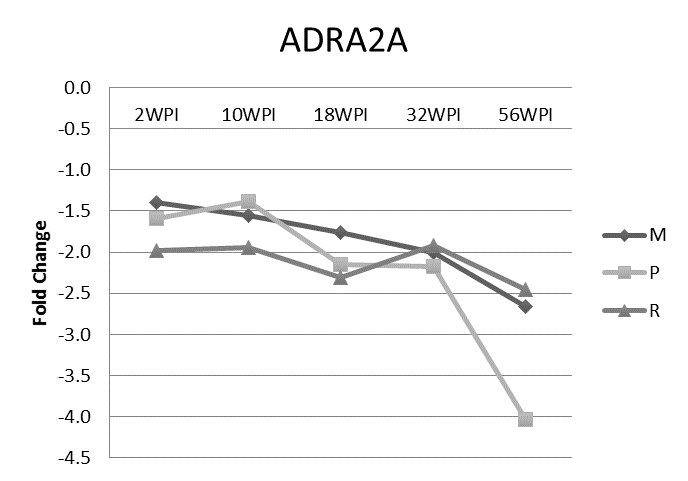

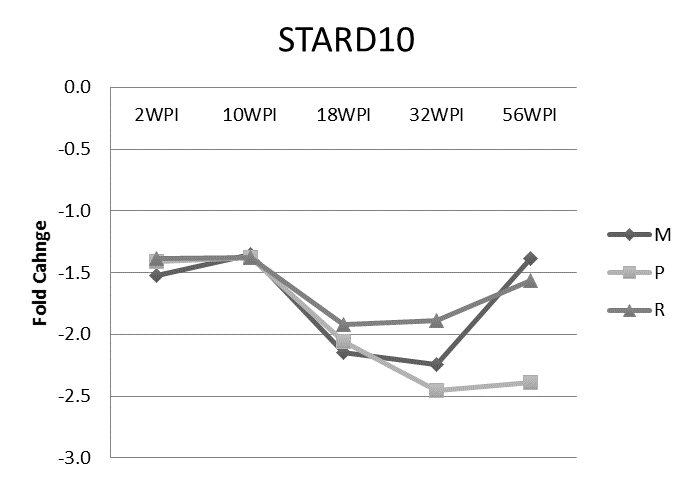

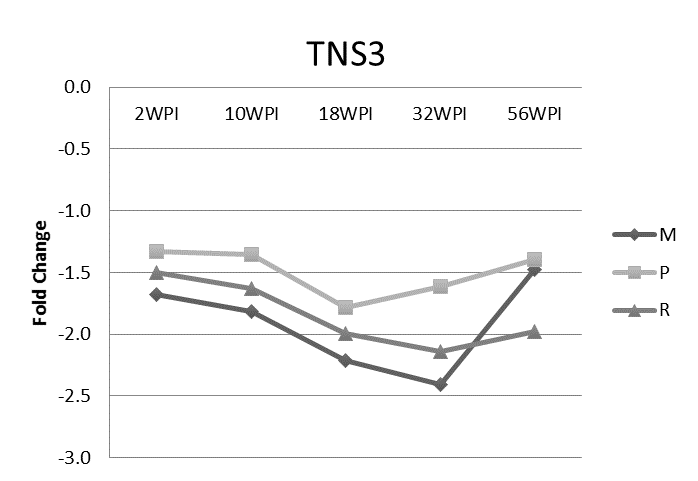

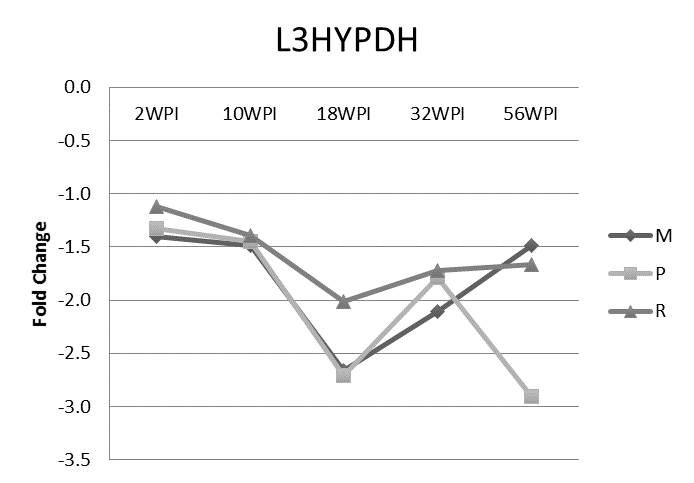

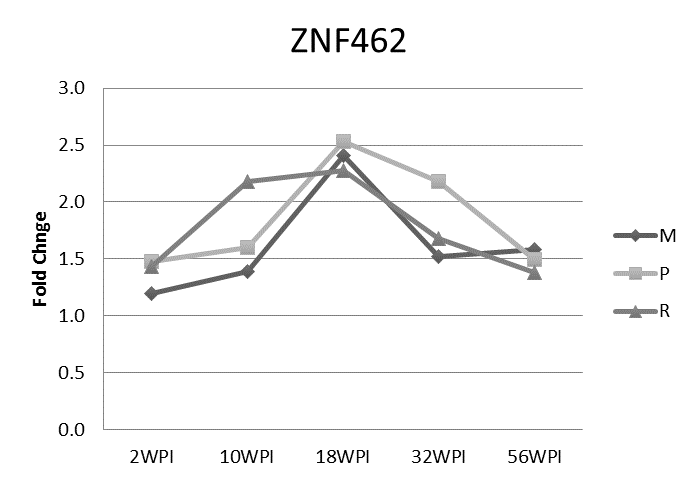

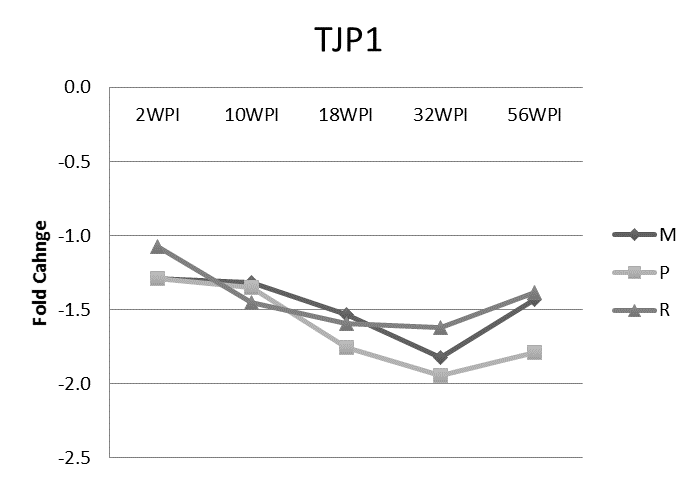

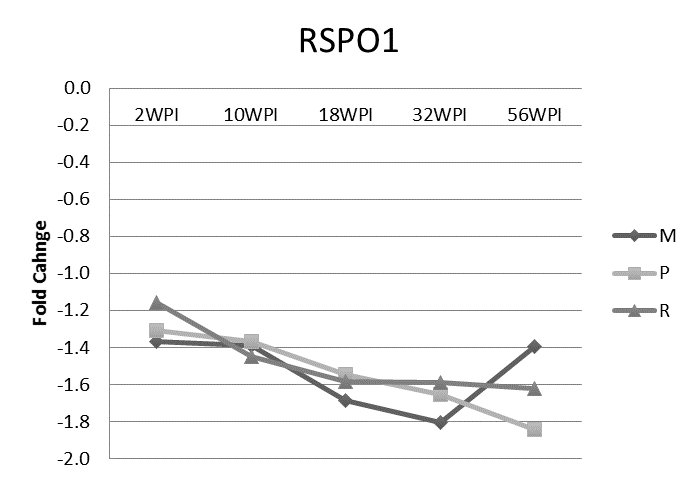

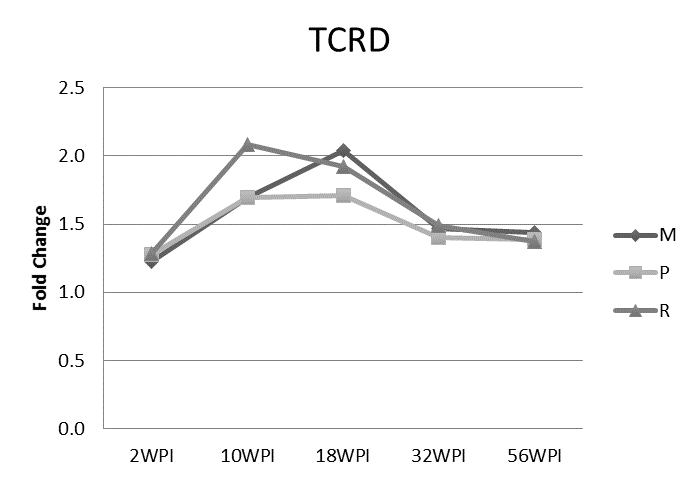

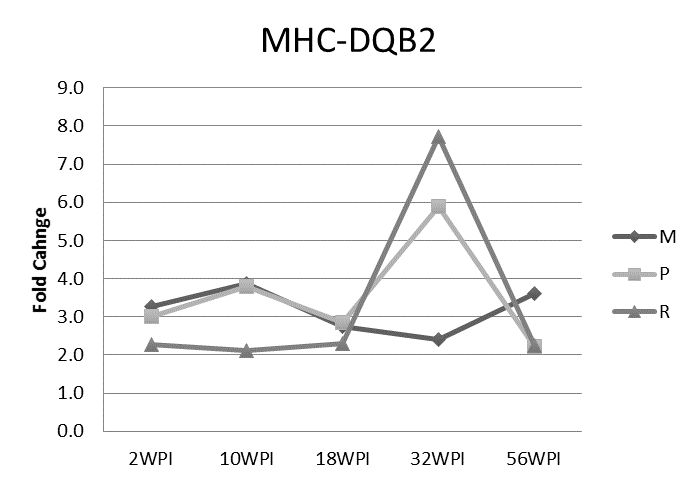


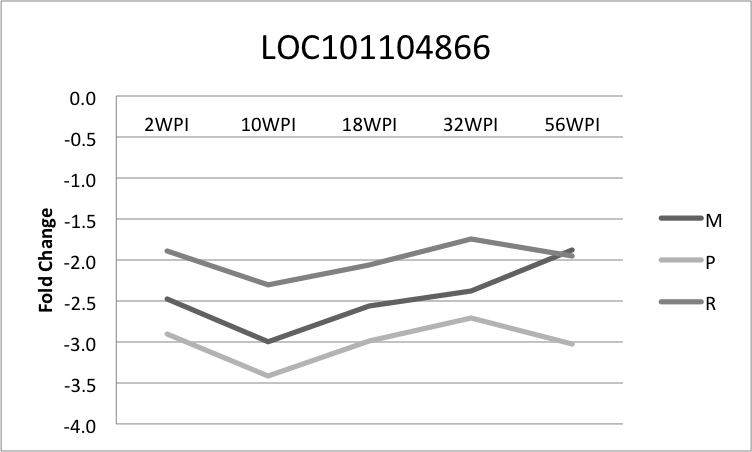


Supplementary Figure 2: Gene expression kinetics of 11 genes similarly changed in all three cohorts. A separate ANOVA was performed to identify genes changed for each cohort within each timepoint as a separate function to the ANOVA performed to identify genes consistently changed across all timepoints with relevance to the cohorts (multibacillary, paucibacillary and resilient). A cross check was performed to verify findings between the two ANOVA experiments.

Supplementary Table 1. List of all genes meeting the criteria for consistent differential expression (FDR ≤0.05 and a FC ≥1.4) across all five sampling times associated with each of the defined groups (multibacillary, paucibacillary or resilient) in comparison to the unexposed control Merino sheep. *Ovis aries* gene symbols and accession numbers matching the Bovine probesets are listed and where there was variation in the Bovine and Ovine sequence gene symbol assignment, the alternate gene symbol is noted. Gene unique to a specific defined group are *italicised* and genes whose expression was similarly changed across all three defined groups are **bold**.

| **Bovine Probeset ID** | **Bovine Gene** | **Ovine Gene** | **Ovine accession** | **Gene Title** | **Multibacillary** | | **Paucibacillary** | | **Resilient** | |
| --- | --- | --- | --- | --- | --- | --- | --- | --- | --- | --- |
|  |  |  |  |  | **p-value** | **FC** | **p-value** | **FC** | **p-value** | **FC** |
| *Bt.10106.1.S1_at* |  | *TRIM9* | *XM_004010522* | *tripartite motif containing 9* |  |  | *7.74E-07* | *1.5* |  |  |
| Bt.10212.1.S1_at |  | PHLDA1 | XM_012150230 | pleckstrin homology-like domain, family A |  |  | 1.52E-03 | -1.4 | 2.43E-05 | -1.4 |
| *Bt.103.1.S1_at* |  | *BPI* | *NM_001306124* | *bactericidal/permeability-increasing protein* |  |  | *9.70E-06* | *1.7* |  |  |
| *Bt.1031.1.S1_at* |  | *CTSH* | *XM_012099136* | *cathepsin H* |  |  | *1.40E-07* | *1.4* |  |  |
| **Bt.10479.2.A1_at** | **CMTM3** | **KRT28** | **XM_004012873.3** | **keratin 28, type I** | **1.77E-03** | **-1.5** | **4.90E-05** | **-1.8** | **5.93E-06** | **-1.7** |
| Bt.10479.3.S1_at | CMTM3 | SMOC2 | XM_012183283 | SPARC related modular calcium binding 2 |  |  | 4.87E-06 | -1.8 | 7.65E-05 | -1.5 |
| *Bt.10875.1.A1_at* |  | *SMIM14* | *XM_012179819* | *small integral membrane protein 14* | *4.05E-08* | *-1.4* |  |  |  |  |
| *Bt.10916.1.S1_at* |  | *NIPA2* | *XM_015093289* | *non imprinted in Prader-Willi/Angelman syndrome 2* |  |  | *4.90E-08* | *-1.5* |  |  |
| Bt.11057.1.S1_at |  | SHISA3 | XM_015096480 | shisa family member 3 | 8.96E-05 | 1.6 | 4.76E-05 | 1.7 |  |  |
| *Bt.11159.1.S1_at* |  | *JUN* | *XM_004002020* | *jun proto-oncogene* | *6.92E-05* | *-1.4* |  |  |  |  |
| *Bt.11415.1.A1_at* | *PRDM11* | *LOC101111903* | *XM_012116933* | *PR domain containing 11* |  |  | *4.17E-04* | *-1.4* |  |  |
| Bt.11580.1.S1_at |  | IGF2BP3 | XM_012176518 | insulin-like growth factor 2 mRNA binding protein 3 | 7.34E-06 | -1.5 | 1.82E-04 | -1.4 |  |  |
| *Bt.11769.1.A1_at* |  | *EID3* | *XM_004006686* | *EP300 interacting inhibitor of differentiation 3* |  |  |  |  | *1.41E-06* | *1.4* |
| Bt.11904.1.S1_at |  | ACSL6 | XM_012178147 | acyl-CoA synthetase long-chain family member 6 | 1.12E-02 | -1.6 |  |  | 1.47E-03 | -1.7 |
| Bt.12048.1.S1_at |  | FAM213A | XM_012168470 | family with sequence similarity 213, member A | 2.04E-02 | 1.4 | 3.95E-02 | 1.4 |  |  |
| *Bt.12176.1.A1_at* | *LOC100335205* | *LOC101122689 (TCRG)* | *XM_015095362* | *T-cell receptor gamma chain C region C10.5* | *1.62E-04* | *1.4* |  |  |  |  |
| Bt.12304.1.S1_at |  | ISG15 | NM_001009735 | ISG15 ubiquitin-like modifier | 2.47E-02 | -1.6 |  |  | 4.58E-02 | -1.4 |
| Bt.12504.1.S1_at |  | CLU | XM_015109143 | clusterin | 3.04E-02 | 1.4 | 4.24E-02 | 1.4 |  |  |
| *Bt.12685.1.S1_at* |  | *MYH11* | *XM_015104094* | *myosin, heavy chain 11, smooth muscle* |  |  | *1.02E-07* | *-1.5* |  |  |
| *Bt.12805.1.S1_at* |  | *PLBD1* | *XM_004006839* | *phospholipase B domain containing 1* |  |  | *2.30E-03* | *1.4* |  |  |
| *Bt.12825.1.S1_at* |  | *ACTA2* | *XM_012160428* | *actin, alpha 2, smooth muscle, aorta* |  |  | *1.46E-02* | *1.4* |  |  |
| *Bt.12906.2.S1_at* |  | *IGK* | *AY734683* | *Ig kappa chain constant region* |  |  | *8.02E-04* | *-1.7* |  |  |
| *Bt.13075.1.S1_at* |  | *HNF4A* | *XM_012188075* | *hepatocyte nuclear factor 4, alpha* |  |  |  |  | *3.68E-03* | *1.4* |
| *Bt.13184.1.S1_at* |  | *LOC105616215* | *XM_012184864* | *multidrug resistance-associated protein 4-like* |  |  | *5.68E-02* | *-2.9* |  |  |
| Bt.13330.2.A1_at |  | PDK4 | XM_004007738 | pyruvate dehydrogenase kinase, isozyme 4 | 2.59E-04 | 1.5 | 3.12E-05 | 1.7 |  |  |
| *Bt.13510.1.S1_at* | *BOLA-DOB* | *LOC101109219* | *XM_012112137* | *MHC class II, DO beta* | *5.10E-05* | *-1.5* |  |  |  |  |
| *Bt.13534.1.S1_at* | *PLA2G16* | *LOC101120283* | *XM_012102083* | *HRAS-like suppressor 3* |  |  | *3.80E-04* | *1.4* |  |  |
| *Bt.13546.1.A1_at* |  | *PARM1* | *XM_012135548* | *prostate androgen-regulated mucin-like protein 1* |  |  | *1.26E-05* | *1.5* |  |  |
| *Bt.1358.1.S1_at* |  | *PDLIM1* | *XM_004020055* | *PDZ and LIM domain 1* | *5.65E-04* | *1.5* |  |  |  |  |
| *Bt.13769.1.S1_at* |  | *MAP2* | *XM_012144751* | *microtubule-associated protein 2* |  |  |  |  | *5.09E-03* | *-1.5* |
| *Bt.13789.1.A1_at* | *CD14* | *DNAH7* | *XM_012141002* | *dynein, axonemal, heavy chain 7* |  |  | *2.52E-03* | *1.4* |  |  |
| *Bt.14256.1.A1_at* | *LOC616903* | *LOC101116026* | *XR_001026281* | *protein CutA homolog* | *2.71E-04* | *-1.4* |  |  |  |  |
| **Bt.1476.1.S1_at** |  | **ADRA2A** | **XM_015103417** | **adrenoceptor alpha 2A** | **1.37E-03** | **-1.8** | **7.19E-04** | **-2.1** | **1.25E-05** | **-2.1** |
| *Bt.15419.1.S1_at* |  | *GCA* | *XM_012166321* | *grancalcin, EF-hand calcium binding protein* | *1.83E-05* | *-1.5* |  |  |  |  |
| *Bt.15435.1.A1_at* | *LOC101904175* | *KLHL14* | *XM_015103607* | *kelch like family member 14* |  |  | *2.38E-04* | *1.4* |  |  |
| *Bt.15758.1.S1_at* |  | *PTGS2* | *NM_001009432* | *prostaglandin-endoperoxide synthase 2 (prostaglandin G/H synthase and cyclooxygenase)* |  |  |  |  | *7.58E-05* | *-1.5* |
| *Bt.1577.1.S1_at* |  | *C1QA* | *XM_004005142* | *complement component 1, q subcomponent, A chain* | *1.45E-03* | *-1.5* |  |  |  |  |
| Bt.15802.1.S1_at |  | KLF5 | XM_004012186 | Kruppel-like factor 5 (intestinal) | 1.01E-04 | -1.8 |  |  | 9.95E-03 | -1.4 |
| Bt.15802.2.S1_at |  | KLF5 | XM_012137639 | Kruppel-like factor 5 (intestinal) | 3.67E-05 | -1.8 |  |  | 2.22E-03 | -1.4 |
| Bt.15915.1.S1_at | IPO11 | LRRC70 | XM_012096865 | importin 11 | 1.34E-09 | 1.6 |  |  | 1.02E-10 | 1.6 |
| *Bt.16036.1.S1_at* |  | *SH3BGRL2* | *XM_012130283* | *SH3 domain binding glutamate-rich protein like 2* |  |  | *1.14E-02* | *1.4* |  |  |
| *Bt.16055.2.S1_a_at* |  | *TAGLN* | *XM_004016051* | *transgelin* | *5.03E-03* | *1.5* |  |  |  |  |
| Bt.16201.1.S1_at |  | S100A9 | XM_012132125 | S100 calcium binding protein A9 | 1.93E-03 | -1.6 |  |  | 2.81E-03 | -1.5 |
| *Bt.16520.1.A1_at* | *CES1* | *LOC101116336* | *XM_012189803* | *liver carboxylesterase 1* | *1.22E-03* | *-1.6* |  |  |  |  |
| *Bt.17368.1.A1_at* |  | *P2RY14* | *XM_012101408* | *purinergic receptor P2Y, G-protein coupled, 14* | *3.41E-04* | *-1.4* |  |  |  |  |
| *Bt.17968.1.S1_at* |  | *PSMD8* | *XM_012190368* | *26S proteasome non-ATPase regulatory subunit 8* |  |  | *5.95E-02* | *-1.5* |  |  |
| *Bt.1807.1.A1_at* |  | *PAK3* | *XM_015105100* | *p21 protein (Cdc42/Rac)-activated kinase 3* | *7.32E-04* | *1.5* |  |  |  |  |
| *Bt.18089.1.S1_at* |  | *TES* | *NM_001195318* | *testin LIM domain protein* |  |  | *2.50E-07* | *-1.6* |  |  |
| *Bt.18208.1.A1_at* |  | *ZBTB32* | *XM_015099961* | *zinc finger and BTB domain containing 32* |  |  | *3.22E-07* | *-1.7* |  |  |
| *Bt.18420.2.A1_at* |  | *EPB41L5* | *XM_012140185* | *erythrocyte membrane protein band 4.1 like 5* |  |  | *1.33E-04* | *1.5* |  |  |
| *Bt.18483.1.A1_at* |  | *METTL24* | *XM_015097348* | *methyltransferase like 24* |  |  | *3.54E-06* | *1.4* |  |  |
| *Bt.18571.3.A1_at* |  | *DLGAP4* | *XM_015099858* | *discs, large (Drosophila) homolog-associated protein 4* | *4.44E-04* | *1.4* |  |  |  |  |
| *Bt.18623.1.A1_at* |  | *MAPRE2* | *XM_004020434* | *microtubule-associated protein, RP/EB family, member 2* |  |  | *1.94E-02* | *1.4* |  |  |
| *Bt.18685.1.A1_at* |  | *DTX1* | *XM_015101665* | *deltex 1, E3 ubiquitin ligase* |  |  | *2.52E-04* | *-1.4* |  |  |
| **Bt.1920.1.S1_at** |  | **STARD10** | **XM_012155230** | **StAR-related lipid transfer (START) domain containing 10** | **9.61E-08** | **-1.6** | **1.31E-07** | **-1.7** | **2.36E-07** | **-1.5** |
| **Bt.1920.2.S1_at** |  | **STARD10** | **XM_015100804** | **StAR-related lipid transfer (START) domain containing 10** | **1.38E-06** | **-1.7** | **9.40E-07** | **-1.9** | **3.18E-06** | **-1.6** |
| *Bt.19204.1.S1_at* |  | *UGGT2* | *XM_012184872* | *UDP-glucose glycoprotein glucosyltransferase 2* | *1.35E-03* | *-1.7* |  |  |  |  |
| *Bt.1922.1.S1_at* | *CLIC5* | *LOC106990969* | *XR_001434161* | *uncharacterized* |  |  | *2.09E-03* | *1.5* |  |  |
| Bt.19405.1.A1_at |  | P2RY13 |  | purinergic receptor P2Y, G-protein coupled, 13 | 3.45E-03 | -1.4 |  |  | 3.03E-06 | -1.6 |
| *Bt.19503.1.A1_at* |  | *PARD3B* | *XM_004004847* | *par-3 family cell polarity regulator beta* |  |  | *3.70E-04* | *1.4* |  |  |
| Bt.196.1.S1_at |  | S100A13 | XM_004022907 | S100 calcium binding protein A13 | 9.67E-09 | 1.4 | 1.15E-05 | 1.4 |  |  |
| *Bt.1978.13.S1_at* |  | *TCRB* | *AF030023* | *T-cell receptor beta chain OTCR23* |  |  |  |  | *1.06E-06* | *1.5* |
| *Bt.19795.1.S1_at* | *BREH1* | *LOC105614501* | *XM_012173338* | *retinyl ester hydrolase type 1* |  |  | *4.40E-03* | *1.4* |  |  |
| *Bt.19797.1.A1_at* |  | *LOC101118184* | *XM_012171593* | *docosahexaenoic acid omega-hydroxylase CYP4F3-like* | *1.53E-03* | *-1.5* |  |  |  |  |
| Bt.19901.1.A1_at |  | SMIM14 | XM_012179819 | small integral membrane protein 14 | 1.14E-07 | -1.6 | 1.32E-04 | -1.4 |  |  |
| *Bt.20035.2.A1_at* |  | *CXXC5* | *XM_015095695* | *CXXC finger protein 5* |  |  | *1.28E-07* | *-1.5* |  |  |
| *Bt.20151.1.S1_at* |  | *DHRS7* | *XM_004010693* | *dehydrogenase/reductase (SDR family) member 7* |  |  |  |  | *6.03E-04* | *-1.4* |
| *Bt.20431.2.S1_at* |  | *MXI1* | *XM_004020185* | *MAX interactor 1, dimerization protein* | *3.07E-10* | *-1.5* |  |  |  |  |
| *Bt.20431.3.S1_at* |  | *MXI1* | *XM_012128387* | *MAX interactor 1, dimerization protein* | *9.35E-09* | *-1.4* |  |  |  |  |
| *Bt.20593.2.S1_at* |  | *PPIC* | *XM_004008676* | *peptidylprolyl isomerase C (cyclophilin C)* |  |  | *1.09E-04* | *1.4* |  |  |
| *Bt.209.1.S1_at* | *LYZ2* | *LOC443320* | *M32493* | *lysozyme 1b* |  |  | *2.45E-04* | *-2.3* |  |  |
| *Bt.209.1.S1_x_at* | *LYZ2* | *LOC443320* | *AH008120* | *lysozyme C-1-like* |  |  | *1.72E-04* | *-2.5* |  |  |
| *Bt.209.2.S1_a_at* |  | *LYZ3* | *XM_015093786* | *lysozyme 3a precursor (* |  |  | *1.73E-03* | *-1.7* |  |  |
| Bt.21027.2.S1_at | LOC104975777 | SPIRE1 | XM_015103675 | spire-type actin nucleation factor 1 | 1.63E-06 | -1.4 |  |  | 3.22E-08 | -1.5 |
| *Bt.21072.1.A1_at* | *ZSCAN23* | *ZSCAN23* | *XM_015102547* | *zinc finger and SCAN domain containing 23* | *3.19E-07* | *1.4* |  |  |  |  |
| Bt.21116.1.S1_at |  | SELENBP1 | XM_012181117 | selenium binding protein 1 | 3.67E-02 | 1.4 | 5.47E-02 | 1.5 |  |  |
| *Bt.21133.1.S1_at* |  | *CAPS2* | *XM_012173940* | *calcyphosine 2, transcript variant X7* | *1.49E-02* | *-1.4* |  |  |  |  |
| *Bt.21227.1.A1_at* |  | *WLS* | *XM_004002069* | *wntless Wnt ligand secretion mediator* | *7.65E-03* | *-1.4* |  |  |  |  |
| *Bt.2129.2.S1_at* |  | *VCAN* | *XM_015096182* | *versican* |  |  | *5.57E-03* | *1.5* |  |  |
| *Bt.21301.1.S1_a_at* |  | *RTN4IP1* | *XM_012182690* | *reticulon 4 interacting protein 1* |  |  | *2.57E-04* | *-1.4* |  |  |
| *Bt.21409.1.A1_at* |  | *COL11A1* | *XM_004002245* | *collagen, type XI, alpha 1* |  |  | *4.29E-03* | *-1.4* |  |  |
| *Bt.21468.1.S1_at* | *DEXI* | *LOC101106819* | *XM_012134280* | *dexamethasone-induced protein* |  |  | *6.41E-11* | *-1.5* |  |  |
| *Bt.21518.1.S1_at* |  | *SIK1* | *XM_015107276* | *salt inducible kinase 1* |  |  | *8.53E-07* | *-1.5* |  |  |
| *Bt.21620.1.A1_at* |  | *JADE3* | *XM_004022142* | *jade family PHD finger 3* |  |  | *2.28E-05* | *1.6* |  |  |
| Bt.21979.1.S1_at |  | CXCR6 | XM_004018516 | chemokine (C-X-C motif) receptor 6 | 1.17E-09 | 1.7 | 5.82E-06 | 1.6 |  |  |
| *Bt.222.1.S1_at* |  | *CRYAB* | *AY819023* | *crystallin, alpha B* | *6.74E-03* | *1.4* |  |  |  |  |
| *Bt.22303.2.S1_at* |  | *KIAA0040* | *XM_012187581* | *KIAA0040 ortholog* |  |  | *4.22E-05* | *-1.4* |  |  |
| *Bt.22390.1.S1_at* |  | *HPS3* | *XM_012168482* | *Hermansky-Pudlak syndrome 3* |  |  | *3.51E-03* | *1.4* |  |  |
| *Bt.22415.2.A1_at* | *SIGLEC14* | *LOC101102213* | *XM_004022966* | *sialic acid-binding Ig-like lectin 14* |  |  |  |  | *5.28E-05* | *-1.4* |
| Bt.22430.1.A1_at |  | CYBB | XM_004021996 | cytochrome b-245, beta polypeptide | 1.06E-09 | -1.4 |  |  | 2.91E-11 | -1.4 |
| *Bt.22526.1.S1_at* |  | *HSPB8* | *XM_012110114* | *heat shock 22kDa protein 8* |  |  | *6.69E-04* | *-1.4* |  |  |
| Bt.22767.1.S1_at | BOLA-NC1 | LOC101104866 | XM_015091331 | MHC class I alpha chain BL3-7 variant x16 | 3.19E-03 | -2.0 | 6.81E-03 | -2.1 |  |  |
| *Bt.22771.1.S1_at* |  | *RAB31* | *XM_015103657* | *RAB31, member RAS oncogene family* | *1.02E-06* | *-1.5* |  |  |  |  |
| *Bt.22852.1.S1_at* |  | *MXRA7* | *XM_012109644* | *matrix-remodelling associated 7* |  |  | *1.00E-03* | *-1.5* |  |  |
| *Bt.22857.1.S1_at* |  | *TRIB2* | *XM_012164685* | *tribbles pseudokinase 2* |  |  | *3.29E-07* | *-1.4* |  |  |
| *Bt.22867.1.S1_x_at* | *BOLA-DQA1* | *LOC101108696* | *NM_001308592* | *MHC class II DQ haplotype D alpha chain like* |  |  |  |  | *2.88E-02* | *1.8* |
| Bt.22867.2.A1_at | BOLA-DQA1 | MHC OVAR-DQA2 | M93433 | MHC class II DQ alpha 2.1 | 1.43E-02 | -2.1 |  |  | 9.91E-05 | -2.9 |
| *Bt.22869.1.S1_at* |  | *FABP5* | *NM_001145180* | *fatty acid binding protein 5* | *5.67E-05* | *1.6* |  |  |  |  |
| *Bt.23094.1.A1_at* | *AKR1C4* | *LOC101109111* | *XM_012172256* | *dihydrodiol dehydrogenase 3* |  |  | *8.87E-05* | *1.6* |  |  |
| *Bt.23227.1.S1_at* |  | *MXD1* | *XM_004005810* | *MAX dimerization protein 1* | *6.40E-03* | *-1.5* |  |  |  |  |
| Bt.23296.1.S1_at | LOC100848815 | MHC-DQB2 | EU176819 | MHC class II DQ beta 2 | 5.23E-02 | 1.6 |  |  | 2.79E-02 | 1.6 |
| Bt.23418.1.S1_at |  | FN1 | XM_004004910 | fibronectin 1 |  |  | 4.49E-06 | -1.8 | 6.50E-05 | -1.5 |
| *Bt.2345.1.S1_at* |  | *PLD4* | *XM_015101837* | *phospholipase D family, member 4* |  |  | *2.01E-06* | *-1.5* |  |  |
| *Bt.23506.2.S1_a_at* |  | *NTPCR* | *XM_004021356* | *nucleoside-triphosphatase, cancer-related* |  |  | *3.21E-07* | *-1.4* |  |  |
| *Bt.23514.1.S1_at* |  | *A2M* | *XM_012175446* | *alpha-2-macroglobulin* |  |  |  |  | *1.28E-04* | *-1.5* |
| Bt.23734.1.A1_at |  | PAWR | XM_015094551 | PRKC, apoptosis, WT1, regulator |  |  | 1.94E-03 | -1.4 | 6.43E-06 | -1.5 |
| **Bt.2399.1.S1_at** |  | **TNS3** | **XM_012176955** | **tensin 3** | **4.44E-09** | **-1.9** | **3.14E-03** | **-1.4** | **4.56E-10** | **-1.8** |
| *Bt.2424.1.S1_at* |  | *DPYD* | *XM_004002217* | *dihydropyrimidine dehydrogenase* |  |  | *1.75E-06* | *1.6* |  |  |
| *Bt.24404.1.A1_at* |  | *EPB41L3* | *XM_012103550* | *erythrocyte membrane protein band 4.1-like 3* |  |  |  |  | *9.31E-08* | *1.6* |
| *Bt.24438.1.S1_a_at* |  | *CSF3R* | *XM_004003483* | *colony stimulating factor 3 receptor (granulocyte)* | *1.76E-02* | *-1.4* |  |  |  |  |
| *Bt.24630.1.S1_at* |  | *SEPT10* | *XM_015094376* | *septin 10* |  |  | *8.00E-04* | *1.4* |  |  |
| *Bt.24630.2.S1_at* |  | *SEPT10* | *XM_015094376* | *septin 10* |  |  | *4.77E-04* | *1.4* |  |  |
| *Bt.24795.1.A1_at* |  | *IFIT2* | *XM_004020282* | *interferon-induced protein with tetratricopeptide repeats 2* | *1.28E-03* | *-1.7* |  |  |  |  |
| Bt.24855.2.S1_at |  | TNFSF13B | DQ152930 | tumor necrosis factor (ligand) superfamily, member 13b | 3.70E-05 | -1.5 |  |  | 4.99E-06 | -1.5 |
| Bt.24928.1.S1_at |  | FILIP1L | XM_004002872 | filamin A interacting protein 1-like | 3.55E-06 | -1.5 | 1.86E-04 | -1.5 |  |  |
| *Bt.24929.1.A1_at* |  | *TRMT10B* | *XM_004004242* | *tRNA methyltransferase 10 homolog B* |  |  |  |  | *2.74E-06* | *-1.5* |
| Bt.24933.1.S1_at |  | RARRES1 | XM_004003212 | retinoic acid receptor responder (tazarotene induced) 1 | 1.22E-05 | 2.4 | 1.64E-03 | 2.1 |  |  |
| **Bt.24972.1.A1_at** |  | **L3HYPDH** | **XM_015097099** | **L-3-hydroxyproline dehydratase (trans-)** | **3.14E-07** | **-1.7** | **2.20E-07** | **-1.9** | **5.81E-06** | **-1.5** |
| *Bt.2529.1.S1_at* |  | *MT2A* | *X07975* | *metallothionein 2A* |  |  | *6.27E-07* | *1.4* |  |  |
| *Bt.25616.1.S1_at* |  | *GPR27* | *XM_012121606* | *G protein-coupled receptor 27* |  |  | *5.58E-05* | *1.4* |  |  |
| **Bt.25937.1.A1_at** |  | **ZNF462** | **XM_012114291** | **zinc finger protein 462** | **3.71E-03** | **1.5** | **9.58E-04** | **1.7** | **4.69E-06** | **1.7** |
| Bt.26155.1.A1_at |  | NPL | XM_012136392 | N-acetylneuraminate pyruvate lyase | 1.16E-02 | 1.4 | 1.24E-02 | 1.5 |  |  |
| Bt.26174.1.A1_at |  | BCL2L15 | XM_004002344 | BCL2-like 15 | 2.35E-05 | -1.4 |  |  | 1.07E-08 | -1.5 |
| Bt.26259.1.A1_at |  | ZNF462 | XM_012114291 | zinc finger protein 462 |  |  | 2.10E-03 | 1.5 | 5.20E-06 | 1.6 |
| *Bt.26803.1.A1_at* |  | *TES* | *NM_001195318* | *testin LIM domain protein* |  |  | *8.35E-08* | *-1.7* |  |  |
| Bt.26920.2.A1_a_at |  | XK | XM_015104848 | X-linked Kx blood group | 1.09E-02 | 1.4 | 1.53E-02 | 1.5 |  |  |
| Bt.26937.1.S1_at |  | LY86 | XM_012101335 | lymphocyte antigen 86-like /// lymphocyte antigen 86 | 2.35E-09 | -1.5 | 5.26E-06 | -1.4 |  |  |
| Bt.26950.1.A1_at |  | SLC39A12 | XM_015099602 | solute carrier family 39 (zinc transporter), member 12 |  |  | 2.80E-04 | -1.8 | 2.49E-05 | -1.7 |
| *Bt.26991.2.S1_a_at* | *C3H1orf162* | *C1H1orf162* | *XM_004002319* | *chromosome 1 open reading frame* | *8.57E-03* | *-1.5* |  |  |  |  |
| **Bt.27008.1.A1_at** |  | **TJP1** | **XM_012098602** | **tight junction protein 1** | **7.39E-07** | **-1.5** | **9.95E-07** | **-1.6** | **6.16E-07** | **-1.4** |
| *Bt.27292.1.S1_at* |  | *LOC101107030* | *XM_015097997* | *multidrug resistance-associated protein 4-like* |  |  | *2.49E-03* | *3.0* |  |  |
| *Bt.27343.1.A1_at* |  | *RIMS1* | *XM_012184227* | *regulating synaptic membrane exocytosis 1* |  |  | *3.78E-06* | *1.4* |  |  |
| Bt.27456.1.A1_at | ELAVL3 | LOC105609456 | XM_012177934 | tyrosine-protein phosphatase non-receptor type 23-like | 1.44E-05 | -1.7 |  |  | 3.16E-04 | -1.4 |
| **Bt.27743.2.A1_at** |  | **RSPO1** | **XM_015091653** | **R-spondin 1** | **9.59E-07** | **-1.5** | **3.52E-06** | **-1.5** | **2.69E-08** | **-1.5** |
| *Bt.27901.1.S1_at* |  | *MAGI1* | *XM_015102112* | *membrane associated guanylate kinase, WW and PDZ domain containing 1* | *1.36E-05* | *1.5* |  |  |  |  |
| Bt.28004.2.S1_a_at |  | TCRD | AJ809504 | partial trdv1.37 gene for T-cell receptor delta | 9.52E-03 | 1.6 |  |  | 6.16E-04 | 1.7 |
| Bt.28022.1.A1_s_at |  | MHC Ovar-I | LT984574 | MHC class I Ovar-N) allele 25:01 | 3.49E-06 | -2.7 | 1.88E-02 | -1.7 |  |  |
| Bt.2815.1.S1_at |  | LXN | XM_004003214 | latexin | 6.96E-03 | 1.5 | 4.75E-02 | 1.4 |  |  |
| *Bt.28271.1.A1_at* |  | *MXD1* | *XM_004005810* | *MAX dimerization protein 1* | *3.51E-04* | *-1.5* |  |  |  |  |
| *Bt.2831.1.S1_at* |  | *EXOSC5* | *XM_004015274* | *exosome component 5* |  |  | *4.59E-07* | *-1.5* |  |  |
| *Bt.28340.1.S1_at* |  | *SLC38A9* | *XM_004016981* | *solute carrier family 38 member 9* | *4.57E-03* | *-1.5* |  |  |  |  |
| *Bt.28378.1.S1_at* |  | *AKAP1* | *XM_015098294* | *A kinase anchoring protein 1* |  |  | *5.79E-08* | *-1.4* |  |  |
| *Bt.28431.1.S1_at* |  | *PARD3B* | *XM_012157627* | *par-3 family cell polarity regulator beta* |  |  | *1.38E-03* | *1.4* |  |  |
| *Bt.28444.1.S1_at* |  | *TCRB* | *AF030008* | *T-cell receptor beta chain OTCR27* |  |  | *4.35E-05* | *-1.5* |  |  |
| Bt.28515.1.A1_at |  | CSNK1G3 | XM_012178215 | casein kinase 1, gamma 3 | 4.57E-06 | -1.6 |  |  | 2.88E-08 | -1.6 |
| **Bt.28732.1.S1_at** |  | **TCRD** | **XM_015096889** | **T-cell receptor delta chain C region** | **8.19E-06** | **1.5** | **1.69E-03** | **1.4** | **2.26E-08** | **1.6** |
| Bt.2892.1.S1_at |  | FABP7 | XM_004011152 | fatty acid binding protein 7 | 4.96E-02 | -1.6 |  |  | 1.10E-02 | -1.7 |
| Bt.29005.1.A1_at |  | RAD51AP1 | XM_012129372 | RAD51-associated protein 1 | 1.51E-03 | -1.6 |  |  | 4.20E-05 | -1.7 |
| *Bt.29053.1.A1_at* |  | *GPR135* | *XM_015097100* | *G protein-coupled receptor 13* |  |  |  |  | *2.96E-02* | *-1.4* |
| Bt.2913.1.A1_at |  | CRYM | XM_012129871 | crystallin, mu | 3.05E-04 | 1.4 |  |  | 2.42E-08 | 1.7 |
| Bt.29214.1.A1_at |  | IGF2BP3 | XM_012176518 | insulin-like growth factor 2 mRNA binding protein 3 | 2.41E-06 | -1.6 | 7.21E-05 | -1.6 |  |  |
| Bt.29689.1.S1_s_at |  | TCRD | XM_015090491 | T-cell receptor delta chain C region | 1.56E-05 | 1.5 |  |  | 2.46E-07 | 1.5 |
| *Bt.29692.1.S1_at* |  | *TCRD* | *AJ868218* | *partial trdv1.5 gene for T cell receptor delta precursor, exons 1-2* |  |  |  |  | *1.82E-08* | *1.4* |
| **Bt.29814.1.S1_at** | **BoLA** | **LOC101104866** | **XM_015091331** | **MHC class I alpha chain BL3-7 variant x16** | **2.75E-06** | **-2.4** | **7.55E-07** | **-3.0** | **3.79E-05** | **-2.0** |
| Bt.29824.1.S1_s_at | BOLA | MHC Ovar-I | LT984574 | MHC class I Ovar-N) allele 25:01 | 5.08E-03 | -4.2 | 5.45E-02 | -3.1 |  |  |
| *Bt.2988.1.S1_at* |  | *GNG11* | *NM_001139445* | *guanine nucleotide binding protein (G protein), gamma 11* |  |  |  |  | *2.79E-04* | *-1.5* |
| *Bt.3014.1.A1_at* |  | *PDGFC* | *XM_015101543* | *platelet derived growth factor C* |  |  | *2.26E-06* | *1.4* |  |  |
| Bt.3046.2.S1_at |  | FCN1 | XM_012117952 | ficolin 1 | 1.73E-02 | 1.6 | 1.52E-05 | 2.9 |  |  |
| *Bt.3210.1.S1_at* |  | *CTNNA1* | *XM_015096061* | *catenin alpha 1* |  |  | *3.31E-05* | *1.5* |  |  |
| *Bt.3220.1.S1_at* |  | *CRYL1* | *XM_004012309* | *crystallin, lambda 1* |  |  | *8.88E-03* | *1.7* |  |  |
| *Bt.3289.1.S1_at* |  | *MTA3* | *XM_012114991* | *metastasis associated 1 family, member 3* |  |  | *1.71E-05* | *-1.6* |  |  |
| *Bt.3311.1.S1_at* |  | *---* |  | *---* | *1.31E-04* | *-1.4* |  |  |  |  |
| *Bt.350.1.S1_at* |  | *MHC-DQB1* | *XM_012173129* | *MHC class II DQ beta 0101* | *1.47E-02* | *1.5* |  |  |  |  |
| **Bt.350.1.S1_s_at** |  | **MHC-DQB2** | **EU176819** | **MHC class II DQ beta domain** | **1.57E-02** | **3.1** | **2.76E-02** | **3.4** | **1.21E-02** | **2.9** |
| *Bt.350.1.S1_x_at* |  | *OVAR-DQB1* | *LT837701* | *MHC class II DQ beta 0101* | *5.88E-03* | *1.5* |  |  |  |  |
| *Bt.3537.1.S1_at* |  | *SULT1A1* | *XM_012104345* | *sulfotransferase family, cytosolic, 1A, phenol-preferring, member 1* |  |  | *2.91E-04* | *1.4* |  |  |
| *Bt.357.1.S1_at* |  | *S100A12* | *XM_012132131* | *S100 calcium binding protein A12* | *4.54E-03* | *-1.5* |  |  |  |  |
| *Bt.3595.1.S1_at* |  | *MGP* | *XM_004006833* | *matrix Gla protein* |  |  |  |  | *6.54E-03* | *-1.4* |
| Bt.367.1.S1_at |  | OLR1 | XM_012165940 | oxidized low density lipoprotein (lectin-like) receptor 1 |  |  | 6.73E-03 | 1.9 | 1.91E-03 | -1.8 |
| *Bt.3698.1.S1_at* |  | *BEX4* | *XM_004022455* | *brain expressed, X-linked 4* |  |  | *5.72E-02* | *1.4* |  |  |
| Bt.3805.1.S1_at | BOLA-N | MHCI | M34673 | MHC class I domain | 4.96E-04 | 1.6 | 2.20E-02 | 1.4 |  |  |
| *Bt.3949.1.S2_at* |  | *PFKFB2* | *XM_004013577* | *6-phosphofructo-2-kinase/fructose-2,6-biphosphatase 2* |  |  | *5.89E-05* | *2.0* |  |  |
| *Bt.405.1.S1_at* |  | *FST* | *M63123* | *follistatin* | *2.03E-05* | *1.4* |  |  |  |  |
| *Bt.4063.1.S1_at* |  | *PLA2G7* | *XM_012114122* | *phospholipase A2, group VII* |  |  | *4.32E-04* | *1.6* |  |  |
| *Bt.4067.1.S1_at* |  | *MARCKS* | *XM_015097338* | *myristoylated alanine-rich protein kinase C substrate* | *8.22E-04* | *-1.5* |  |  |  |  |
| *Bt.4102.2.S1_a_at* | *NPPC* | *CNP* | *AF037467* | *natriuretic peptide C* |  |  | *1.84E-06* | *-1.4* |  |  |
| *Bt.4208.1.S1_at* |  | *CXCR1* | *XM_004004919* | *chemokine (C-X-C motif) receptor 1* | *2.33E-02* | *-1.5* |  |  |  |  |
| *Bt.4336.1.S1_at* |  | *CFD* | *XM_015093908* | *complement factor D (adipsin)* |  |  | *1.45E-02* | *1.4* |  |  |
| *Bt.435.1.S1_at* |  | *TIMP2* | *XM_015098286* | *TIMP metallopeptidase inhibitor 2* |  |  |  |  | *7.75E-06* | *-1.5* |
| *Bt.4357.1.S1_at* |  | *TSPAN7* | *XM_004022003* | *tetraspanin 7* |  |  | *1.84E-06* | *1.7* |  |  |
| *Bt.4621.1.S1_a_at* |  | *SMTN* | *XM_015101717* | *smoothelin* | *9.27E-03* | *1.4* |  |  |  |  |
| *Bt.4751.2.S1_a_at* | *BOLA-DQA2* | *MHCII-DQA2* | *HG798790* | *MHC class II DQ alpha 2 allele 01:02:01* |  |  | *1.04E-05* | *-1.7* |  |  |
| Bt.4758.1.S1_at |  | FABP3 | NM_001267884 | fatty acid binding protein 3 |  |  | 1.88E-08 | -1.9 | 1.27E-06 | -1.5 |
| Bt.4762.1.S1_at | BOLA-NC1 | LOC101113217 | XM_015105290 | MHC class I alpha BL3-7-like | 1.47E-04 | 1.6 | 9.18E-03 | 1.5 |  |  |
| *Bt.4898.1.S1_at* |  | *BASP1* | *XM_015101334* | *brain abundant, membrane attached signal protein 1* | *4.42E-03* | *-1.5* |  |  |  |  |
| *Bt.5129.1.S1_a_at* |  | *NNAT* | *XM_012114753* | *neuronatin* |  |  |  |  | *1.12E-04* | *-1.7* |
| *Bt.5132.1.S1_at* |  | *DDAH2* | *XM_012163418* | *dimethylarginine dimethylaminohydrolase 2* | *5.34E-12* | *-1.7* |  |  |  |  |
| *Bt.514.1.S1_at* |  | *DNAJC6* | *XM_012169019* | *DnaJ (Hsp40) homolog, subfamily C, member 6* |  |  | *5.31E-07* | *-1.5* |  |  |
| *Bt.5301.1.S1_at* |  | *THBS1* | *XM_015096932* | *thrombospondin 1* |  |  |  |  | *1.24E-04* | *-1.5* |
| Bt.5302.1.S1_a_at |  | MRVI1 | XM_012095776 | murine retrovirus integration site 1 homolog | 1.85E-02 | 1.4 | 4.53E-02 | 1.4 |  |  |
| Bt.5304.1.S1_at |  | GSTM3 | XM_012124095 | glutathione S-transferase mu 3 | 5.38E-04 | 2.2 | 2.17E-03 | 2.3 |  |  |
| *Bt.5324.1.S1_s_at* | *BOLA-N* | *OLA-I* | *M34675* | *MHC class I domain* | *6.99E-07* | *-1.4* |  |  |  |  |
| *Bt.5395.1.S1_a_at* |  | *VCAN* | *XM_015096182* | *versican core protein* |  |  |  |  | *7.69E-04* | *-1.5* |
| *Bt.5408.1.A1_at* |  | *UCHL1* | *XM_004009789* | *ubiquitin C-terminal hydrolase L1* | *2.27E-08* | *1.5* |  |  |  |  |
| *Bt.5499.1.S1_at* |  | *CAPG* | *XM_012169258* | *capping protein (actin filament), gelsolin-like* |  |  | *2.42E-09* | *1.4* |  |  |
| *Bt.5536.1.S1_at* |  | *ITGB5* | *XM_015091733* | *integrin, beta 5* |  |  | *2.79E-05* | *1.4* |  |  |
| *Bt.5598.1.S1_at* |  | *C27H8orf4* | *XM_012125947* | *chromosome 27 open reading frame, human C8orf4* |  |  | *1.21E-02* | *-1.7* |  |  |
| *Bt.5605.1.S1_at* |  | *MARCKS* | *XM_015097338* | *myristoylated alanine-rich protein kinase C substrate* | *3.94E-03* | *-1.5* |  |  |  |  |
| *Bt.5605.2.S1_at* |  | *MARCKS* | *XM_012166905* | *myristoylated alanine-rich protein kinase C substrate* | *2.69E-03* | *-1.6* |  |  |  |  |
| *Bt.5605.3.S1_at* |  | *MARCKS* | *XM_015097338* | *myristoylated alanine-rich protein kinase C substrate* | *4.69E-03* | *-1.5* |  |  |  |  |
| Bt.569.1.S1_at |  | MAP2 | XM_012144751 | microtubule-associated protein 2 |  |  | 3.69E-02 | -1.4 | 2.41E-03 | -1.5 |
| *Bt.5878.1.A1_at* |  | *SDS* | *XM_012167605* | *serine dehydratase* | *7.76E-03* | *-1.5* |  |  |  |  |
| *Bt.5878.2.S1_at* |  | *SDS* | *XM_012167605* | *serine dehydratase* | *1.31E-02* | *-1.5* |  |  |  |  |
| *Bt.6069.1.S1_at* | *AKAP2* | *AKAP2* | *XM_012113877* | *A kinase anchor protein 2* |  |  | *9.81E-05* | *1.5* |  |  |
| Bt.640.1.A1_at | LOC101906812 | LOC105601928 | XM_012172675 | multidrug resistance-associated protein 4-like |  |  | 2.07E-02 | 1.4 | 2.57E-04 | 1.5 |
| *Bt.6406.1.S2_at* | *CEBPD* | *CEBPB* | *XM_012134346* | *CCAAT/enhancer binding protein (C/EBP), delta* |  |  | *7.09E-04* | *1.5* |  |  |
| *Bt.6705.1.S1_at* |  | *TBC1D8* | *XM_015094489* | *TBC1 domain family, member 8* | *4.50E-03* | *-1.4* |  |  |  |  |
| *Bt.6824.1.A1_at* |  | *TET2* | *XM_015096388* | *tet methylcytosine dioxygenase 2* | *2.01E-02* | *1.6* |  |  |  |  |
| Bt.7056.3.A1_a_at |  | HBG | AH001245 | hemoglobin, gamma | 5.28E-04 | -2.5 | 4.39E-04 | 3.0 |  |  |
| *Bt.7056.3.A1_x_at* | *HBG* | *LOC101106199* | *XM_004016241* | *hemoglobin fetal subunit beta* |  |  | *8.92E-04* | *1.6* |  |  |
| Bt.7056.5.A1_x_at | HBG | LOC101106199 | XM_012164565 | hemoglobin fetal subunit beta | 1.20E-02 | -1.6 | 2.69E-04 | 2.4 |  |  |
| *Bt.736.1.A1_at* |  | *TGM1* | *XM_004010301* | *transglutaminase 1* | *3.39E-03* | *-1.5* |  |  |  |  |
| Bt.7394.1.A1_at | PTPLA | HACD1 | NM_001009443 | 3-hydroxyacyl-CoA dehydratase 1 | 5.21E-05 | -1.5 | 1.56E-04 | -1.5 |  |  |
| *Bt.7467.1.S1_at* |  | *KCTD12* | *XM_015098226* | *potassium channel tetramerization domain containing 12* |  |  | *3.21E-05* | *1.4* |  |  |
| *Bt.7727.1.S1_at* |  | *KANK2* | *XR_001437661* | *KN motif and ankyrin repeat domains 2* |  |  | *2.04E-06* | *1.4* |  |  |
| *Bt.7728.1.A1_at* |  | *DCBLD2* | *XM_012188531* | *discoidin, CUB and LCCL domain containing 2* |  |  | *1.78E-04* | *1.4* |  |  |
| *Bt.8166.3.A1_s_at* |  | *CXXC5* | *XM_012150650* | *CXXC finger protein 5* |  |  | *6.95E-07* | *-1.4* |  |  |
| *Bt.83.1.S1_a_at* |  | *DNAJC5* | *XR_001435530* | *DnaJ (Hsp40) homolog, subfamily C, member 5* | *4.49E-05* | *1.4* |  |  |  |  |
| Bt.8976.1.S1_at |  | TCF7L2 | XM_015103420 | transcription factor 7-like 2 | 7.35E-05 | -1.6 |  |  | 2.64E-04 | -1.5 |
| *Bt.8990.1.S1_at* |  | *NAGA* | *XM_012158576* | *N-acetylgalactosaminidase, alpha-* |  |  | *7.31E-09* | *1.5* |  |  |
| *Bt.9202.1.S1_at* |  | *FGL2* | *XM_012142958* | *fibrinogen-like 2* | *9.45E-04* | *-1.7* |  |  |  |  |
| *Bt.9262.1.A1_at* |  | *SPIB* | *XM_015100076* | *Spi-B transcription factor (Spi-1/PU.1 related)* |  |  | *1.09E-05* | *-1.4* |  |  |
| *Bt.9360.1.S1_at* |  | *S100A8* | *XM_004002523* | *S100 calcium binding protein A8* | *1.18E-03* | *-1.6* |  |  |  |  |
| *Bt.958.1.A1_at* |  | *TNFAIP6* | *XM_012149818* | *tumor necrosis factor, alpha-induced protein 6* | *3.70E-03* | *-1.7* |  |  |  |  |
| *Bt.9594.1.S1_at* |  | *ITGB3* | *XM_012186283* | *integrin, beta 3 (platelet glycoprotein IIIa, antigen CD61)* |  |  | *1.39E-04* | *1.8* |  |  |
| *Bt.9605.1.S1_at* |  | *MS4A8* | *XM_012155435* | *membrane-spanning 4-domains, subfamily A, member 8* | *1.52E-02* | *-1.4* |  |  |  |  |
| *Bt.9722.1.S1_at* |  | *TMEM154* | *XM_012097354* | *transmembrane protein 154* |  |  | *9.92E-03* | *-1.4* |  |  |
| *Bt.9774.1.S1_a_at* | *C10H15orf48* | *C7H15orf48* | *XM_004010648* | *chromosome 7 open reading frame, human C15orf48* |  |  |  |  | *1.50E-03* | *-1.4* |
| Bt.9807.1.S1_at |  | GPNMB | XM_004007790 | glycoprotein (transmembrane) nmb | 1.27E-05 | -2.1 |  |  | 5.79E-04 | -1.7 |
| Bt.9807.2.S1_at |  | GPNMB | XM_004007790 | glycoprotein (transmembrane) nmb | 1.68E-05 | -1.6 |  |  | 3.64E-04 | -1.4 |
| *Bt.981.2.S1_at* |  | *CLIC4* | *XM_004005110* | *chloride intracellular channel 4* |  |  | *2.54E-03* | *1.4* |  |  |

Supplementary Table 2. qPCR results

| Affymetrix ID / Gene name | Comparison | Fold Change qRT-PCR | P-value |
| --- | --- | --- | --- |
| Bt.103.1.S1_at | Multibacillary/control | 1.9 | ns |
| BPI | Recovered/control | -1.2 | ns |
|  | Paucibacillary/control | 9.5 | 9.51E-06 |
|  |  |  |  |
| Bt.16520.1.A1_at | Multibacillary/control | -5.9 | 6.22E-03 |
| CES1 | Recovered/control | 2.9 | ns |
|  | Paucibacillary/control | 3.7 | ns |
|  |  |  |  |
| Bt.18571.1.S1_at | Multibacillary/control | 4.1 | 2.35E-04 |
| DLGAP4 | Recovered/control | 1.0 | ns |
|  | Paucibacillary/control | 1.4 | ns |
|  |  |  |  |
| Bt.20295.1.A1_at | Multibacillary/control | 3.0 | 1.61E-04 |
| FST | Recovered/control | -1.024 | ns |
|  | Paucibacillary/control | 1.2 | ns |
|  |  |  |  |
| Bt.3046.2.S1_at | Multibacillary/control | 7.2 | 3.51E-04 |
| FCN1 | Recovered/control | 1.0 | ns |
|  | Paucibacillary/control | 3.9 | 1.26E-05 |
|  |  |  |  |
| Bt.9594.1.S1_at | Multibacillary/control | 1.4 | ns |
| ITGB3 | Recovered/control | 2.0 | ns |
|  | Paucibacillary/control | 1.5 | 8.17E-04 |
|  |  |  |  |
| Bt.2815.1.S1_at | Multibacillary/control | 6.3 | 1.23E-03 |
| LXN | Recovered/control | 1.4 | ns |
|  | Paucibacillary/control | 3.1 | 4.68E-03 |
|  |  |  |  |
| Bt.1920.1.S1_at | Multibacillary/control | -7 | 1.47E-05 |
| STARD10 | Recovered/control | -5.6 | 1.53E-04 |
|  | Paucibacillary/control | -3.8 | 7.13E-05 |
|  |  |  |  |
| Bt.20151.1.S1_at | Multibacillary/control | 1.6 | ns |
| DHRS7 | Recovered/control | -5.5 | 6.16E-04 |
|  | Paucibacillary/control | 1.5 | ns |
|  |  |  |  |
| Bt.24929.1.A1_at | Multibacillary/control | 1.4 | ns |
| TRMT10B | Recovered/control | -1.5 | 3.25E-03 |
|  | Paucibacillary/control | 1.5 | ns |
|  |  |  |  |
| Bt.2988.1.S1_at | Multibacillary/control | 1.7 | ns |
| GNG11 | Recovered/control | -2.1 | 2.39E-03 |
|  | Paucibacillary/control | 1.6 | ns |
|  |  |  |  |
| Bt.9774.1.S1_a_at | Multibacillary/control | 1.6 | ns |
| C10H15orf48 | Recovered/control | -6.0 | 2.92E-03 |
|  | Paucibacillary/control | 1.5 | ns |
|  |  |  |  |
| Bt.350.1.S1_s_at | Multibacillary/control | 7.3 | 7.37E-05 |
| BLA-DQB | Recovered/control | 2.1 | 6.50E-05 |
|  | Paucibacillary/control | 2.2 | 5.69E-04 |

Table 3. Ingenuity Pathway Analysis eligible differentially regulated focus genes – Multibacillary compared to Control

| Gene Symbol | Affymetrix ID^a^ | p-value | Fold Change |
| --- | --- | --- | --- |
| HLA-B | Bt.28022.1.A1_s_at | 3.49E-06 | -2.7 |
| HBD | Bt.7056.3.A1_a_at | 5.28E-04 | -2.5 |
| GPNMB | Bt.9807.1.S1_at | 1.27E-05 | -2.1 |
| TNS3 | Bt.2399.1.S1_at | 4.44E-09 | -1.9 |
| KLF5 | Bt.15802.1.S1_at | 1.01E-04 | -1.8 |
| FGL2 | Bt.9202.1.S1_at | 9.45E-04 | -1.7 |
| IFIT2 | Bt.24795.1.A1_at | 1.28E-03 | -1.7 |
| TNFAIP6 | Bt.958.1.A1_at | 3.70E-03 | -1.7 |
| STARD10 | Bt.1920.2.S1_at | 1.38E-06 | -1.7 |
| DDAH2 | Bt.5132.1.S1_at | 5.34E-12 | -1.7 |
| ELAVL3 | Bt.27456.1.A1_at | 1.44E-05 | -1.7 |
| IGF2BP3 | Bt.29214.1.A1_at | 2.41E-06 | -1.6 |
| S100A8 | Bt.9360.1.S1_at | 1.18E-03 | -1.6 |
| MARCKS | Bt.5605.2.S1_at | 2.69E-03 | -1.6 |
| CES1 | Bt.16520.1.A1_at | 1.22E-03 | -1.6 |
| S100A9 | Bt.16201.1.S1_at | 1.93E-03 | -1.6 |
| ISG15 | Bt.12304.1.S1_at | 2.47E-02 | -1.6 |
| FABP7 | Bt.2892.1.S1_at | 4.96E-02 | -1.6 |
| ACSL6 | Bt.11904.1.S1_at | 1.12E-02 | -1.6 |
| SMIM14 | Bt.19901.1.A1_at | 1.14E-07 | -1.6 |
| CSNK1G3 | Bt.28515.1.A1_at | 4.57E-06 | -1.6 |
| CXCR2 | Bt.4208.1.S1_at | 2.33E-02 | -1.5 |
| CYP4F2 | Bt.19797.1.A1_at | 1.53E-03 | -1.5 |
| C1orf162 | Bt.26991.2.S1_a_at | 8.57E-03 | -1.5 |
| FILIP1L | Bt.24928.1.S1_at | 3.55E-06 | -1.5 |
| LY86 | Bt.26937.1.S1_at | 2.35E-09 | -1.5 |
| MXD1 | Bt.28271.1.A1_at | 3.51E-04 | -1.5 |
| BASP1 | Bt.4898.1.S1_at | 4.42E-03 | -1.5 |
| C1QA | Bt.1577.1.S1_at | 1.45E-03 | -1.5 |
| CMTM3 | Bt.10479.2.A1_at | 1.77E-03 | -1.5 |
| TNFSF13B | Bt.24855.2.S1_at | 3.70E-05 | -1.5 |
| HACD1 | Bt.7394.1.A1_at | 5.21E-05 | -1.5 |
| SDS | Bt.5878.2.S1_at | 1.31E-02 | -1.5 |
| TGM1 | Bt.736.1.A1_at | 3.39E-03 | -1.5 |
| HLA-DOB | Bt.13510.1.S1_at | 5.10E-05 | -1.5 |
| MXI1 | Bt.20431.2.S1_at | 3.07E-10 | -1.5 |
| RAB31 | Bt.22771.1.S1_at | 1.02E-06 | -1.5 |
| TJP1 | Bt.27008.1.A1_at | 7.39E-07 | -1.5 |
| S100A12 | Bt.357.1.S1_at | 4.54E-03 | -1.5 |
| GCA | Bt.15419.1.S1_at | 1.83E-05 | -1.5 |
| RSPO1 | Bt.27743.2.A1_at | 9.59E-07 | -1.5 |
| Cutal | Bt.14256.1.A1_at | 2.71E-04 | -1.4 |
| JUN | Bt.11159.1.S1_at | 6.92E-05 | -1.4 |
| P2RY14 | Bt.17368.1.A1_at | 3.41E-04 | -1.4 |
| CSF3R | Bt.24438.1.S1_a_at | 1.76E-02 | -1.4 |
| CYBB | Bt.22430.1.A1_at | 1.06E-09 | -1.4 |
| MS4A8 | Bt.9605.1.S1_at | 1.52E-02 | -1.4 |
| TBC1D8 | Bt.6705.1.S1_at | 4.50E-03 | -1.4 |
| P2RY13 | Bt.19405.1.A1_at | 3.45E-03 | -1.4 |
| WLS | Bt.21227.1.A1_at | 7.65E-03 | -1.4 |
| CLU | Bt.12504.1.S1_at | 3.04E-02 | 1.4 |
| DNAJC5 | Bt.83.1.S1_a_at | 4.49E-05 | 1.4 |
| FST | Bt.405.1.S1_at | 2.03E-05 | 1.4 |
| SMTN | Bt.4621.1.S1_a_at | 9.27E-03 | 1.4 |
| MRVI1 | Bt.5302.1.S1_a_at | 1.85E-02 | 1.4 |
| ZSCAN26 | Bt.21072.1.A1_at | 3.19E-07 | 1.4 |
| XK | Bt.26920.2.A1_a_at | 1.09E-02 | 1.4 |
| SELENBP1 | Bt.21116.1.S1_at | 3.67E-02 | 1.4 |
| FAM213A | Bt.12048.1.S1_at | 2.04E-02 | 1.4 |
| NPL | Bt.26155.1.A1_at | 1.16E-02 | 1.4 |
| CRYAB | Bt.222.1.S1_at | 6.74E-03 | 1.4 |
| S100A13 | Bt.196.1.S1_at | 9.67E-09 | 1.4 |
| CRYM | Bt.2913.1.A1_at | 3.05E-04 | 1.4 |
| ZNF462 | Bt.25937.1.A1_at | 3.71E-03 | 1.5 |
| PDK4 | Bt.13330.2.A1_at | 2.59E-04 | 1.5 |
| UCHL1 | Bt.5408.1.A1_at | 2.27E-08 | 1.5 |
| MAGI1 | Bt.27901.1.S1_at | 1.36E-05 | 1.5 |
| LXN | Bt.2815.1.S1_at | 6.96E-03 | 1.5 |
| TAGLN | Bt.16055.2.S1_a_at | 5.03E-03 | 1.5 |
| PDLIM1 | Bt.1358.1.S1_at | 5.65E-04 | 1.5 |
| SHISA3 | Bt.11057.1.S1_at | 8.96E-05 | 1.6 |
| TET2 | Bt.6824.1.A1_at | 2.01E-02 | 1.6 |
| HLA-DQA1 | Bt.23296.1.S1_at | 5.23E-02 | 1.6 |
| FABP5 | Bt.22869.1.S1_at | 5.67E-05 | 1.6 |
| FCN1 | Bt.3046.2.S1_at | 1.73E-02 | 1.6 |
| IPO11 | Bt.15915.1.S1_at | 1.34E-09 | 1.6 |
| CXCR6 | Bt.21979.1.S1_at | 1.17E-09 | 1.7 |
| GSTM3 | Bt.5304.1.S1_at | 5.38E-04 | 2.2 |
| RARRES1 | Bt.24933.1.S1_at | 1.22E-05 | 2.4 |
| HLA-DQB1 | Bt.350.1.S1_s_at | 1.57E-02 | 3.1 |

Affymetrix probe set ID from Bovine GeneChip Array.

Table 4. Ingenuity Pathway Analysis eligible differentially regulated focus genes – Paucibacillary compared to Control

| Gene Symbol | Affymetrix ID^a^ | p-value | Fold Change |
| --- | --- | --- | --- |
| HLA-B | Bt.22767.1.S1_at | 6.81E-03 | -2.1 |
| STARD10 | Bt.1920.2.S1_at | 9.40E-07 | -1.9 |
| FABP3 | Bt.4758.1.S1_at | 1.88E-08 | -1.9 |
| CMTM3 | Bt.10479.2.A1_at | 4.90E-05 | -1.8 |
| SLC39A12 | Bt.26950.1.A1_at | 2.80E-04 | -1.8 |
| C8orf4 | Bt.5598.1.S1_at | 1.21E-02 | -1.7 |
| ZBTB32 | Bt.18208.1.A1_at | 3.22E-07 | -1.7 |
| TES | Bt.26803.1.A1_at | 8.35E-08 | -1.7 |
| MTA3 | Bt.3289.1.S1_at | 1.71E-05 | -1.6 |
| IGF2BP3 | Bt.29214.1.A1_at | 7.21E-05 | -1.6 |
| TJP1 | Bt.27008.1.A1_at | 9.95E-07 | -1.6 |
| HACD1 | Bt.7394.1.A1_at | 1.56E-04 | -1.5 |
| DEXI | Bt.21468.1.S1_at | 6.41E-11 | -1.5 |
| RSPO1 | Bt.27743.2.A1_at | 3.52E-06 | -1.5 |
| MYH11 | Bt.12685.1.S1_at | 1.02E-07 | -1.5 |
| CXXC5 | Bt.20035.2.A1_at | 1.28E-07 | -1.5 |
| NIPA2 | Bt.10916.1.S1_at | 4.90E-08 | -1.5 |
| DNAJC6 | Bt.514.1.S1_at | 5.31E-07 | -1.5 |
| FILIP1L | Bt.24928.1.S1_at | 1.86E-04 | -1.5 |
| EXOSC5 | Bt.2831.1.S1_at | 4.59E-07 | -1.5 |
| PLD4 | Bt.2345.1.S1_at | 2.01E-06 | -1.5 |
| PSMD8 | Bt.17968.1.S1_at | 5.95E-02 | -1.5 |
| MAP2 | Bt.569.1.S1_at | 3.69E-02 | -1.4 |
| DTX1 | Bt.18685.1.A1_at | 2.52E-04 | -1.4 |
| KIAA0040 | Bt.22303.2.S1_at | 4.22E-05 | -1.4 |
| SMIM14 | Bt.19901.1.A1_at | 1.32E-04 | -1.4 |
| TRIB2 | Bt.22857.1.S1_at | 3.29E-07 | -1.4 |
| COL11A1 | Bt.21409.1.A1_at | 4.29E-03 | -1.4 |
| LY86 | Bt.26937.1.S1_at | 5.26E-06 | -1.4 |
| AKAP1 | Bt.28378.1.S1_at | 5.79E-08 | -1.4 |
| TNS3 | Bt.2399.1.S1_at | 3.14E-03 | -1.4 |
| NTPCR | Bt.23506.2.S1_a_at | 3.21E-07 | -1.4 |
| PAWR | Bt.23734.1.A1_at | 1.94E-03 | -1.4 |
| HSPB8 | Bt.22526.1.S1_at | 6.69E-04 | -1.4 |
| NPPC | Bt.4102.2.S1_a_at | 1.84E-06 | -1.4 |
| RTN4IP1 | Bt.21301.1.S1_a_at | 2.57E-04 | -1.4 |
| SPIB | Bt.9262.1.A1_at | 1.09E-05 | -1.4 |
| KANK2 | Bt.7727.1.S1_at | 2.04E-06 | 1.4 |
| RIMS1 | Bt.27343.1.A1_at | 3.78E-06 | 1.4 |
| CTSH | Bt.1031.1.S1_at | 1.40E-07 | 1.4 |
| S100A13 | Bt.196.1.S1_at | 1.15E-05 | 1.4 |
| PLBD1 | Bt.12805.1.S1_at | 2.30E-03 | 1.4 |
| PLA2G16 | Bt.13534.1.S1_at | 3.80E-04 | 1.4 |
| Sult1a1 | Bt.3537.1.S1_at | 2.91E-04 | 1.4 |
| Mapre2 | Bt.18623.1.A1_at | 1.94E-02 | 1.4 |
| PDGFC | Bt.3014.1.A1_at | 2.26E-06 | 1.4 |
| HPS3 | Bt.22390.1.S1_at | 3.51E-03 | 1.4 |
| ITGB5 | Bt.5536.1.S1_at | 2.79E-05 | 1.4 |
| MRVI1 | Bt.5302.1.S1_a_at | 4.53E-02 | 1.4 |
| PPIC | Bt.20593.2.S1_at | 1.09E-04 | 1.4 |
| CD14 | Bt.13789.1.A1_at | 2.52E-03 | 1.4 |
| PARD3B | Bt.28431.1.S1_at | 1.38E-03 | 1.4 |
| CAPG | Bt.5499.1.S1_at | 2.42E-09 | 1.4 |
| KCTD12 | Bt.7467.1.S1_at | 3.21E-05 | 1.4 |
| ACTA2 | Bt.12825.1.S1_at | 1.46E-02 | 1.4 |
| CES1 | Bt.19795.1.S1_at | 4.40E-03 | 1.4 |
| SH3BGRL2 | Bt.16036.1.S1_at | 1.14E-02 | 1.4 |
| CFD | Bt.4336.1.S1_at | 1.45E-02 | 1.4 |
| DCBLD2 | Bt.7728.1.A1_at | 1.78E-04 | 1.4 |
| LXN | Bt.2815.1.S1_at | 4.75E-02 | 1.4 |
| FAM213A | Bt.12048.1.S1_at | 3.95E-02 | 1.4 |
| CLU | Bt.12504.1.S1_at | 4.24E-02 | 1.4 |
| SEPT10 | Bt.24630.2.S1_at | 4.77E-04 | 1.4 |
| VCAN | Bt.2129.2.S1_at | 5.57E-03 | 1.5 |
| SELENBP1 | Bt.21116.1.S1_at | 5.47E-02 | 1.5 |
| TRIM9 | Bt.10106.1.S1_at | 7.74E-07 | 1.5 |
| PALM2-AKAP2 | Bt.6069.1.S1_at | 9.81E-05 | 1.5 |
| XK | Bt.26920.2.A1_a_at | 1.53E-02 | 1.5 |
| CEBPD | Bt.6406.1.S2_at | 7.09E-04 | 1.5 |
| CTNNA1 | Bt.3210.1.S1_at | 3.31E-05 | 1.5 |
| NPL | Bt.26155.1.A1_at | 1.24E-02 | 1.5 |
| CLIC5 | Bt.1922.1.S1_at | 2.09E-03 | 1.5 |
| NAGA | Bt.8990.1.S1_at | 7.31E-09 | 1.5 |
| JADE3 | Bt.21620.1.A1_at | 2.28E-05 | 1.6 |
| PLA2G7 | Bt.4063.1.S1_at | 4.32E-04 | 1.6 |
| CXCR6 | Bt.21979.1.S1_at | 5.82E-06 | 1.6 |
| AKR1C3 | Bt.23094.1.A1_at | 8.87E-05 | 1.6 |
| DPYD | Bt.2424.1.S1_at | 1.75E-06 | 1.6 |
| ZNF462 | Bt.25937.1.A1_at | 9.58E-04 | 1.7 |
| CRYL1 | Bt.3220.1.S1_at | 8.88E-03 | 1.7 |
| PDK4 | Bt.13330.2.A1_at | 3.12E-05 | 1.7 |
| SHISA3 | Bt.11057.1.S1_at | 4.76E-05 | 1.7 |
| TSPAN7 | Bt.4357.1.S1_at | 1.84E-06 | 1.7 |
| BPI | Bt.103.1.S1_at | 9.70E-06 | 1.7 |
| ITGB3 | Bt.9594.1.S1_at | 1.39E-04 | 1.8 |
| OLR1 | Bt.367.1.S1_at | 6.73E-03 | 1.9 |
| PFKFB2 | Bt.3949.1.S2_at | 5.89E-05 | 2.0 |
| RARRES1 | Bt.24933.1.S1_at | 1.64E-03 | 2.1 |
| GSTM3 | Bt.5304.1.S1_at | 2.17E-03 | 2.3 |
| FCN1 | Bt.3046.2.S1_at | 1.52E-05 | 2.9 |
| HBD | Bt.7056.3.A1_a_at | 4.39E-04 | 3.0 |
| HLA-DQB1 | Bt.350.1.S1_s_at | 2.76E-02 | 3.4 |

Affymetrix probe set ID from Bovine GeneChip Array.

Table 5. Ingenuity Pathway Analysis eligible differentially regulated focus genes – Resilient compared to Control

| Gene Symbol | Affymetrix ID^a^ | p-value | Fold Change |
| --- | --- | --- | --- |
| TNS3 | Bt.2399.1.S1_at | 4.56E-10 | -1.8 |
| OLR1 | Bt.367.1.S1_at | 1.91E-03 | -1.8 |
| FABP7 | Bt.2892.1.S1_at | 1.10E-02 | -1.7 |
| CMTM3 | Bt.10479.2.A1_at | 5.93E-06 | -1.7 |
| GPNMB | Bt.9807.1.S1_at | 5.79E-04 | -1.7 |
| NNAT | Bt.5129.1.S1_a_at | 1.12E-04 | -1.7 |
| ACSL6 | Bt.11904.1.S1_at | 1.47E-03 | -1.7 |
| SLC39A12 | Bt.26950.1.A1_at | 2.49E-05 | -1.7 |
| P2RY13 | Bt.19405.1.A1_at | 3.03E-06 | -1.6 |
| CSNK1G3 | Bt.28515.1.A1_at | 2.88E-08 | -1.6 |
| STARD10 | Bt.1920.2.S1_at | 3.18E-06 | -1.6 |
| TIMP2 | Bt.435.1.S1_at | 7.75E-06 | -1.5 |
| MAP2 | Bt.569.1.S1_at | 2.41E-03 | -1.5 |
| A2M | Bt.23514.1.S1_at | 1.28E-04 | -1.5 |
| FABP3 | Bt.4758.1.S1_at | 1.27E-06 | -1.5 |
| S100A9 | Bt.16201.1.S1_at | 2.81E-03 | -1.5 |
| PTGS2 | Bt.15758.1.S1_at | 7.58E-05 | -1.5 |
| GNG11 | Bt.2988.1.S1_at | 2.79E-04 | -1.5 |
| TNFSF13B | Bt.24855.2.S1_at | 4.99E-06 | -1.5 |
| PAWR | Bt.23734.1.A1_at | 6.43E-06 | -1.5 |
| TRMT10B | Bt.24929.1.A1_at | 2.74E-06 | -1.5 |
| VCAN | Bt.5395.1.S1_a_at | 7.69E-04 | -1.5 |
| RSPO1 | Bt.27743.2.A1_at | 2.69E-08 | -1.5 |
| THBS1 | Bt.5301.1.S1_at | 1.24E-04 | -1.5 |
| KLF5 | Bt.15802.2.S1_at | 2.22E-03 | -1.4 |
| ISG15 | Bt.12304.1.S1_at | 4.58E-02 | -1.4 |
| ELAVL3 | Bt.27456.1.A1_at | 3.16E-04 | -1.4 |
| C15orf48 | Bt.9774.1.S1_a_at | 1.50E-03 | -1.4 |
| MGP | Bt.3595.1.S1_at | 6.54E-03 | -1.4 |
| CYBB | Bt.22430.1.A1_at | 2.91E-11 | -1.4 |
| TJP1 | Bt.27008.1.A1_at | 6.16E-07 | -1.4 |
| DHRS7 | Bt.20151.1.S1_at | 6.03E-04 | -1.4 |
| HNF4A | Bt.13075.1.S1_at | 3.68E-03 | 1.4 |
| EID3 | Bt.11769.1.A1_at | 1.41E-06 | 1.4 |
| HLA-DQA1 | Bt.23296.1.S1_at | 2.79E-02 | 1.6 |
| IPO11 | Bt.15915.1.S1_at | 1.02E-10 | 1.6 |
| EPB41L3 | Bt.24404.1.A1_at | 9.31E-08 | 1.6 |
| CRYM | Bt.2913.1.A1_at | 2.42E-08 | 1.7 |
| ZNF462 | Bt.25937.1.A1_at | 4.69E-06 | 1.7 |
| HLA-DQB1 | Bt.350.1.S1_s_at | 1.21E-02 | 2.9 |

^a^ Affymetrix probe set ID from Bovine GeneChip Array.
